# Anti-Inflammatory and Antioxidative Phytogenic Substances against Secret Killers in Poultry: Current Status and Prospects

**DOI:** 10.3390/vetsci10010055

**Published:** 2023-01-14

**Authors:** Shereen Basiouni, Guillermo Tellez-Isaias, Juan D. Latorre, Brittany D. Graham, Victor M. Petrone-Garcia, Hesham R. El-Seedi, Sakine Yalçın, Amr Abd El-Wahab, Christian Visscher, Helen L. May-Simera, Claudia Huber, Wolfgang Eisenreich, Awad A. Shehata

**Affiliations:** 1 Institute of Molecular Physiology, Johannes-Gutenberg University, 55128 Mainz, Germany; 2 Clinical Pathology Department, Faculty of Veterinary Medicine, Benha University, Moshtohor, Toukh 13736, Egypt; 3 Department of Poultry Science, University of Arkansas Agricultural Experiment Station, Fayetteville, AR 72701, USA; 4 Facultad de Estudios Superiores Cuautitlan, Universidad Nacional Autonoma de Mexico (UNAM), Cuautitlan Izcalli 58190, Mexico; 5 Pharmacognosy Group, Department of Pharmaceutical Biosciences, Uppsala University, Biomedical Centre, SE 751 24 Uppsala, Sweden; 6 International Research Center for Food Nutrition and Safety, Jiangsu University, Zhenjiang 212013, China; 7 International Joint Research Laboratory of Intelligent Agriculture and Agri-Products Processing, Jiangsu Education Department, Jiangsu University, Nanjing 210024, China; 8 Department of Animal Nutrition and Nutritional Diseases, Faculty of Veterinary Medicine, Ankara University (AU), 06110 Ankara, Turkey; 9 Institute for Animal Nutrition, University of Veterinary Medicine Hannover, Foundation, Bischofsholer Damm 15, 30173 Hanover, Germany; 10 Department of Nutrition and Nutritional Deficiency Diseases, Faculty of Veterinary Medicine, Mansoura University, Mansoura 35516, Egypt; 11 Structural Biochemistry of Membranes, Bavarian NMR Center, Technical University of Munich (TUM), D-85747 Garching, Germany; 12 Avian and Rabbit Diseases Department, Faculty of Veterinary Medicine, University of Sadat City, Sadat City 32897, Egypt; 13 Research and Development Section, PerNaturam GmbH, An der Trift 8, 56290 Gödenroth, Germany; 14 Prophy-Institute for Applied Prophylaxis, 59159 Bönen, Germany

**Keywords:** poultry, inflammation, oxidative stress, stressors, phytogenic substances

## Abstract

**Simple Summary:**

Chronic stress and inflammation, known also as “secret killers” in animals, can lead to lipid peroxidation, protein oxidation and nitration, DNA damage, and finally apoptosis. This is due to an imbalance between free radical generation and endogenous antioxidant defense, which in turn possess detrimental impacts on the health and performance of animals. In this review, we discuss the mechanistic pathways of oxidative stress and inflammation associated with the main secret killers in poultry, namely heat stress, dysbiosis, leaky gut syndrome, and mycotoxins. Additionally, we shed light on the potential use, challenges, and future prospects of phytogenic bioactive substances in mitigating oxidative stress and inflammation in poultry.

**Abstract:**

Chronic stress is recognized as a secret killer in poultry. It is associated with systemic inflammation due to cytokine release, dysbiosis, and the so-called leaky gut syndrome, which mainly results from oxidative stress reactions that damage the barrier function of the cells lining the gut wall. Poultry, especially the genetically selected broiler breeds, frequently suffer from these chronic stress symptoms when exposed to multiple stressors in their growing environments. Since oxidative stress reactions and inflammatory damages are multi-stage and long-term processes, overshooting immune reactions and their down-stream effects also negatively affect the animal’s microbiota, and finally impair its performance and commercial value. Means to counteract oxidative stress in poultry and other animals are, therefore, highly welcome. Many phytogenic substances, including flavonoids and phenolic compounds, are known to exert anti-inflammatory and antioxidant effects. In this review, firstly, the main stressors in poultry, such as heat stress, mycotoxins, dysbiosis and diets that contain oxidized lipids that trigger oxidative stress and inflammation, are discussed, along with the key transcription factors involved in the related signal transduction pathways. Secondly, the most promising phytogenic substances and their current applications to ameliorate oxidative stress and inflammation in poultry are highlighted.

## 1. Introduction

Mitochondria, commonly referred as the “powerhouse of eukaryotic cells”, are responsible for the production of cellular energy [1]. However, mitochondria are also involved in numerous additional metabolic processes, such as signaling through mitochondrial reactive oxygen species (ROS), hormonal signaling, heme synthesis reactions, steroid synthesis, regulation of membrane permeability, apoptosis-induced cell death, calcium trafficking, and control of cellular metabolism [2,3]. As a result, mitochondrial damage and subsequent malfunction are significant contributing factors to a variety of animal diseases, owing to their influence on cellular metabolism [4,5]. Additionally, ROS can be generated in the cytosol and other cellular compartments, including the plasma membrane, but also the nucleus, peroxisome, endoplasmic reticulum (ER), and Golgi apparatus [6,7,8]. Due to the high contents of polyunsaturated fatty acids (PUFAs) in these membranes [9], lipid peroxidation can occur and, as a result, phospholipids become directly damaged and may also act as a signal for death [10].

Stress, regardless of its source or type (biological, environmental, nutritional, physical, chemical, or psychological), can lead to inflammation and further malicious downstream reactions [11,12,13]. Several synthetic compounds have been developed to significantly lower inflammation, but most of these drugs are accompanied by unwanted side effects, especially when used at higher doses and during long-term therapies. Natural compounds appear to be less compromised by these side effects [14] and, especially in poultry farming, phytogenic feed additives (PFAs) have attracted considerable interest [15]. Generally, the utilization of natural feed additives that contain anti-inflammatory phytochemicals has become very common for the enhancement of productivity, digestive enzymes, nutrient utilization and as an alternative to antibiotics in livestock species and poultry in particular. The phytochemical compounds of interest are diverse in their structures and include polyphenols, flavonoids, terpenoids, alkaloids and plant sterols [16]. In addition to their anti-inflammatory and antioxidant properties, they may also have a number of other effects, including anticancer, antimicrobials, anti-diarrheal, and analgesic actions [17], which in turn enhance the profitability of poultry.

The current review summarizes the most important anti-inflammatory and antioxidant phytogenic compounds and their uses in poultry. Moreover, this review describes the current knowledge of how these compounds affect oxidative stress and inflammation processes, including the key transcription factors involved in signal transduction pathways.

## 2. Oxidative Stress

During normal oxygen metabolism, cells are continually exposed to free radicals and other ROS [18], serving as, for example, signaling molecules involved in homeostasis. Extreme stressors may enhance the levels of ROS, thus leading to lipid peroxidation, cell membrane and DNA damage, and modification of small GTPases [19,20]. In turn, these processes pave the way for chronic stress symptoms.

### 2.1. Reactive Species

The following two types of reactive species are known: (i) ROS that comprise free radicals (lipid peroxyl radicals (ROO^•^), thiyl radicals (^•^RS), superoxide anion radicals (O_2_^•−^), and hydroxyl radicals (HO^•^)), and non-radical species (hydrogen peroxide (H_2_O_2_), single oxygen (^1^O_2_), ozone (O_3_), and lipid peroxides (ROOH)); (ii) reactive nitrogen species (RNS), including free radicals (nitric oxide (NO∙) and nitrogen dioxide (^•^NO_2_)), and the non-radicals (dinitrogen trioxide (N_2_O_3_), dinitrogen tetraoxide (N_2_O_4_), and peroxynitrite (ONOO^−^)). Oxidative stress is also impacted by aggressive metal ions, such as Fe^2+^/Fe^3+^ and Cu^+^/Cu^2+^ [21,22]. These reactive species are primarily produced by the electron transport chain in the mitochondria (the main source) and by the nicotinamide adenine dinucleotide phosphate oxidases (NADPH oxidase or NOX) in the cell membrane, including the seven transmembrane enzymes, NOX1–NOX5, dual oxidase 1 (DUOX1), and DUOX2 [23,24].

### 2.2. Endogenous Antioxidants

**Figure 1:**
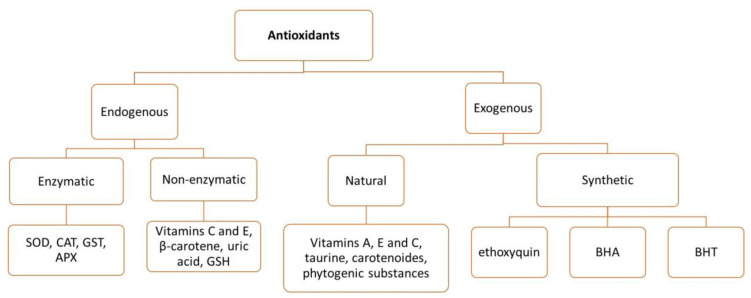
Endogenous and exogenous antioxidants. SOD, superoxide dismutase; CAT, catalase; GST, glutathione S-transferase; APX, ascorbate peroxidase; GSH, glutathione; BHA, butylated hydroxyanisole; BHT, butylated hydroxy toluene.

Endogenous antioxidants have the capacity to donate H atoms to counteract the negative impacts of ROS and RNS [25]. They function at various levels, thereby efficiently limiting the generation of reactive species and scavenging ROS and RNS using non-catalytic and catalytic molecules, such as alpha-tocopherol and ascorbic acid. This can also be accomplished by repairing damaged molecules, by regenerating antioxidants or lipid radicals, to their original state [25,26]. The main enzymatic antioxidants are superoxide dismutase (SOD), catalase (CAT), glutathione reductase (GR), glutathione peroxidase (GPx), glutathione S-transferase (GST), and ascorbate peroxidase (APX) [27]. The classification of antioxidants is illustrated in Figure 1.

The diverse mechanisms of action of these enzymes are well-known and shall not be repeated here [28,29]. As a principle, the nuclear factor erythroid 2-related factor 2 (Nrf2) controls the expression of many antioxidant enzymes. When cells experience oxidative stress, Nrf2 becomes active, translocates to the nucleus, binds to the antioxidant response elements (AREs), and thus activates the genes that code for detoxifying enzymes, such as SOD [30].

Non-enzymatic endogenous antioxidants include vitamins (vitamins C and E), β-carotene, and glutathione (L-glutamyl-L-cysteinylglycine, GSH) that contains a reactive thiol (sulfhydryl) group. Vitamin C, a water-soluble antioxidant, predominantly scavenges oxygen free radicals in the intracellular and extracellular space [31]. It also reacts with reactive vitamin E radicals, converting them to vitamin E [32]. Vitamin E is an antioxidant that prevents free radicals from damaging cell membranes and other fat-soluble substances. Fat-soluble vitamins are the primary defense against oxidant-induced membrane damage. Vitamin E detoxifies peroxyl radicals, which are formed during lipid peroxidation by donating an electron to the antioxidant. Vitamin E demonstrates not only antioxidant actions, but also shields other antioxidants from oxidation. Vitamin E in the most active form, α-tocopherol, is the main cell’s main membrane-bound antioxidant [32,33].

In contrast, GSH is the most significant hydrophilic antioxidant that protects cells from exogenous and endogenous ROS and RNS. When GSH reacts with ROS or other electrophiles, it is oxidized to glutathione disulfide (GSSG). It may then be reduced back to GSH by GR, which uses NAD(P)H as an electron donor. As a result, the GSH/GSSG ratio reflects the oxidative status and can interact with redox partners to keep the cell’s redox balance stable. GSH exhibits antioxidant properties in a variety of ways [34]. By virtue of the action of glutathione peroxidase (GSH-Px), it detoxifies H_2_O_2_ and lipid peroxides. GSH provides an electron to H_2_O_2_ to convert it into H_2_O and O_2_ [35]. GSH-Pxs are also necessary for cell membrane defense against lipid peroxidation. Reduced glutathione transfers protons to membrane lipids, protecting them from oxidative stress [36].

### 2.3. Imbalance between Free Radicals and Antioxidants

An imbalance between free radical production and the level of endogenous antioxidants causes oxidative stress in cells, resulting in lipid peroxidation, protein nitration and oxidation, DNA damage, and finally apoptosis. Under normal redox conditions, various enzyme systems contribute to redox homeostasis in cells by maintaining physiologically important ROS at low levels [37]. Although strong antioxidant induction is linked to Nrf2 when this route is triggered by ROS, this response is restricted because ROS also activate a cell death signaling pathway [38,39]. The increased production of ROS in cells alters or activates numerous intracellular mechanisms that cause the oxidation of DNA, proteins, and membrane lipids. Induced lipid peroxidation by ROS significantly contributes to cell death, including apoptosis [40]. ROS participate in lipid peroxidation, especially the peroxidation of lipids and lipoproteins that are rich in PUFAs. The product of PUFA peroxidation is 4-hydroxynonenal, which further exacerbates mitochondrial dysfunction, impairs cell signaling, and causes further oxidative damage to the cell membranes. By producing ROS, cells are incited to undergo apoptosis via the activation of p53, p38 mitogen-activated protein kinase (MAPK), caspases, and changes in Bcl-2/Bax expression, apoptosis regulators that directly control mitochondrial outer membrane permeabilization [41].

In summary, insufficient levels of antioxidants lead to the accumulation of ROS and RNS, thereby triggering oxidative damage and inflammation [42,43,44]. In this non-physiological state, cells secrete inflammatory cytokines and chemokines, which contribute to attracting other cells to fight against infection and promote tissue regeneration [45]. ROS are also known to activate the nuclear transcription factor (NF-κB) [46], a multi-directional transcriptional regulatory protein that is closely related to various physiological and pathological processes, such as oxidative stress, inflammation, immune response, cell proliferation, transformation, and apoptosis. NF-κB is a key target in receptor-independent hypothalamic micro-inflammation [47] that is associated with intracellular organelle stress, including endoplasmic reticulum stress [48] and defective autophagy [49]. Numerous crucial physiological processes are regulated by NF-κB. However, it has been demonstrated that excessive NF-κB activation increases the risk of disease, while NF-κB suppression is associated with risk reduction [50].

### 2.4. Biomarkers of Oxidative Stress

Assessment of oxidative stress based on direct ROS and RNS measurement is difficult due to the short half-life of ROS [51]. However, there are several indirect biomarkers, which are as follows: (i) biomarkers for lipid peroxidation, such as malondialdehyde (MDA), thiobarbituric acid reactive substances, isoprostanes, and 4-hydroxyalkenals, including 4-hydroxynonenal [52]. It was suggested that isoprostanes are the best markers for lipid peroxidation because they are unique end products of the peroxidation of PUFAs [53]. (ii) Biomarkers for protein oxidation, such as carbonyl moieties in the side chains of amino acids, also exist. These carbonyl moieties can be detected by ELISA techniques, Western blot, or FPLC/HPLC [54]. (iii) Finally, there are also biomarkers for DNA oxidation, such as 8-hydroxy-2′-deoxyguanosine. DNA damage can also be evaluated by comet assays [55].

## 3. Factors for Oxidative Stress and Inflammation in Poultry: Secret Killers

In animal farming, a variety of environmental, nutritional, microbiological, and management factors contribute to oxidative stress. These stressors can be termed as “secret killers”, since they multiply in malignant states in animals [56]. In this section, we focus on the most important factors that are relevant to poultry farming, such as heat stress, dysbiosis and mycotoxins.

During chronic inflammation, an increase in the generation of ROS causes the peroxidation of lipids in cell membranes, as well as mitochondrial and other endomembranes, finally leading to cell death [57]. When these membranes are damaged over time, it is not surprising that multiple cells and organs of an organism are affected [58]. Animal studies [59,60] have established that the complex interactions among diet ingredients, the gut microbiome, the nervous system, the immune system, and the endocrine system are crucial for metabolic and gastrointestinal health. Any disturbances in this delicate equilibrium, such as chronic oxidative stress, result in mitochondrial dysfunction, with its severe impacts upon the immune system and microbiota (see below).

Ninety percent of pathological problems are linked to intestinal chronic inflammation [61]. Disbalance of the gut microbiota has negative effects on the health and biology of metazoans because the gut integrity, biology, metabolism, nutrition, immunity, and neuroendocrine system are all dependent on a healthy microbiota [62,63,64,65,66,67], which is in constant interaction with the microbiota–brain–gut axis. In conclusion, it is justified to qualify oxidative stress and intestinal inflammation as the “secret killers” in animal farming, especially in poultry farming [56,62,68].

### 3.1. Heat Stress

High temperature is one of the most challenging stressors associated with poultry production [69,70]. It is a serious problem for poultry reared in tropical and subtropical regions, as well as in temperate climate zones, including central and eastern Europe [71]. Heat stress occurs when the ambient temperature exceeds the animal’s thermoneutral zone, and the animal’s physiological capacity to disperse heat through sweating, breathing, or panting fails to prevent a rise in body temperature [72]. Chickens are susceptible to high ambient temperatures due to their feathers, lack of skin sweat glands, and high production of heat, unlike mammals. Chickens lose excess heat by panting to prevent the increase in their body temperature [73]. Heat stress causes several adverse effects on the intestinal mucus layer, tight junctions, enteric immune system, and the antioxidant system [74], which are as follows: (i) a decrease in the size of mucin layers. Heat stress reduces the amount of goblet cells, as well as the expression and secretion of mucins, leading to the thinning of mucin protective layers [75]. As a result, their resistance to opportunistic bacteria decreases and these come in more contact with the intestinal epithelial cells. The following effects are also caused by heat stress: (ii) disruption of tight junctions, as heat stress alters the expression of tight junction protein constituents, such as occludin (OCLN), various claudins (CLDN) and zonula occludens (ZO)-1, -2 and -3 [75,76]; (iii) intestinal barrier dysfunction, as the intestinal hyperpermeability is increased [77,78,79,80]; (iv) endotoxemia and systemic inflammation, which results from the translocation of opportunistic bacteria, endotoxins and lipopolysaccharides (LPS), leading to an increase in pro-inflammatory mediators, such as interleukins (IL-1β, IL-6) and tumor necrosis factor-α (TNF-α) [81]; v) hepatic and hypothalamic inflammation, which mainly results from the translocation of microbial-associated molecular patterns, such as LPS [82]; (vi) redox imbalance between the pro- and antioxidants in favor of pro-oxidants. Heat stress is a key contributor to systemic oxidative stress by increasing the levels of pro-oxidants (e.g., ROS). Several studies have revealed that heat stress leads to higher cellular energy demand, promoting the generation of ROS in the mitochondria [83,84]. Consequently, oxidative stress occurs in multiple tissues, leading to cell apoptosis or necrosis [85].

**Figure 2:**
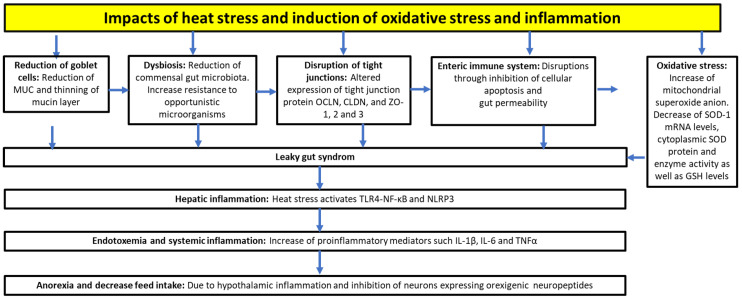
Impacts of heat stress on physiological functions, and induction of inflammation and oxidative stress. OCLN, occludin; CLDN, claudins; ZO, zonula occludens; TLR4, toll-like receptor 4; NF-κB, nuclear factor-kappa B; IL, interleukin; TNFα, tumor necrosis factor α; SOD, superoxide dismutase 1; GSH, glutathione.

In summary, heat-induced oxidative stress disrupts the intestinal barrier and alters many cellular processes. Thus, the presence of ROS increases the intestinal permeability, which facilitates the translocation of bacteria and their molecular patterns (e.g., LPS) from the gut (leaky gut syndrome) [37] (see also Figure 2).

### 3.2. Dysbiosis

**Figure 3:**
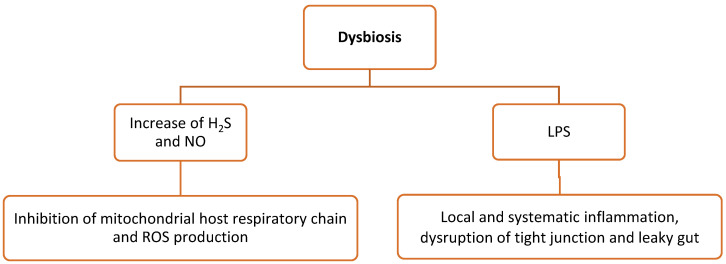
Microbial metabolites during dysbiosis-mediated oxidative stress and inflammation. H_2_S, hydrogen sulfide; ROS, reactive oxygen species; IL, interleukins; LPS, lipopolysaccharides.

Poultry production relies heavily on the animals’ intestinal health and intestinal function to maximize nutrient uptake and growth, which in turn are associated with animal performance. Their gut microbiota mainly consists of bacteria, fungi, and protozoa. As a result of commensal bacteria, intestinal epithelial cells create ROS, which serve as second messengers in cellular signaling. Tight junctions between intestinal epithelial cells form a barrier and prevent the invasion of microorganisms into the host organism [86]. Dysbiosis refers to the alteration in the composition of the gut microbiota with an imbalanced host–microbe relationship [87,88]. As a result, this can lead to increasing amounts of microbial metabolites (see below) that mediate oxidative stress and inflammation (Figure 3).

More specifically, ROS are generated in the gut epithelial cells by several ROS stressors that disrupt the redox balance and cause inflammation, which are as follows [59]: (i) NO is produced by the gut microbiota in the intestinal tissues via the conversion of nitrite and nitrate [89]. Excessive production of NO due to dysbiosis generates ROS associated with cellular damages, e.g., due to the inhibition of the host mitochondrial respiratory chain [90]. (ii) Some intestinal bacteria such as *E. coli* produce hydrogen sulfide (H_2_S) in high amounts by the degradation of sulfur-containing peptides and amino acids in the gut. In the case of dysbiosis, the elevated H_2_S concentration inhibits cytochrome oxidase, which in turn inhibits the host mitochondrial respiratory chain and leads to the overexpression of pro-inflammatory factors [91]. However, H_2_S can also be detoxified by the cecal mucosa by converting it into thiosulfate, which is subsequently converted by ROS into tetrathionate, serving as an electron acceptor for salmonellae, as an example. As a result, a new nutrient niche in the gut is shaped by supporting the growth of more pathogenic bacteria and, thus, increasing dysbiosis and gut inflammation [92,93]. (iii) The TCA cycle can be stimulated by short-chain fatty acids (SCFAs), particularly butyrate. In addition, SCFAs can promote the production of the signaling hormone GLP-1 and the anti-inflammatory IL-10 cytokines to decrease energy intake [91]. (iv) During dysbiosis, LPS production by Gram-negative bacteria is increased and induces local and systematic inflammation by the stimulation of the intestinal epithelial cells and macrophages. As a result, tight junctions are damaged, leading to leaky gut syndrome [94,95,96,97,98,99].

### 3.3. Mycotoxins

**Figure 4:**
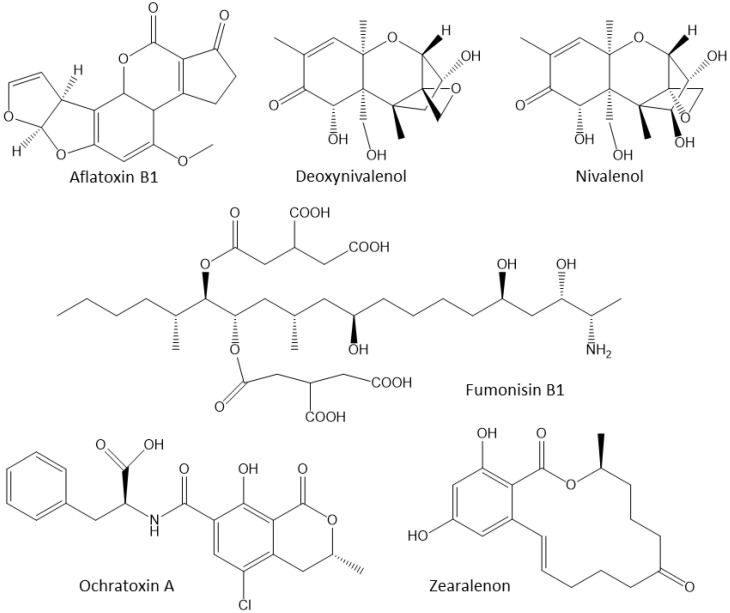
The most common mycotoxins that affect poultry. Aflatoxin B1 (AFB1) and fumonisin B1 (FB1) are polar mycotoxins that are more easily adsorbed by adsorbents than non-polar mycotoxins. Ochratoxin A, T-2 toxin, deoxynivalenol (DON) and zearalenone (ZEN) are non-polar.

Foods, grains, and animal diets are suitable substrates for a wide array of fungi and molds. In particular, molds such as *Aspergillus*, *Fusarium*, and *Penicillium* species produce their own strain-specific mycotoxins as secondary metabolites and the mycotoxin-contaminated diets have to be discarded [100]. Due to significant economic losses, mycotoxins are a global issue. Aflatoxin B1 (AFB1), deoxynivalenol (DON), nivalenol (NIV), fumonisin B1 (FB1), ochratoxin A (OTA), and zearalenone (ZEN) are the main mycotoxins [101,102,103] (Figure 4).

In poultry farming, mycotoxins reduce feed intake, feed efficiency, growth performance, immunity, and hatchability [104,105]. The toxins increase mortality, organ damage, carcinogenicity, teratogenicity, and decrease egg production. On a molecular level, mycotoxins induce the generation of ROS, and thereby contribute to lipid peroxidation [106]. They also alter cellular redox signaling, antioxidant status, and membrane integrity [107]. Mycotoxins, particularly aflatoxin, suppress the intracellular levels of antioxidants Nrf2, SOD, GPx and CAT [108,109], and, thus, increase lipid peroxidation and reduce GSH levels [110,111]. The main intracellular endogenous antioxidants and pro-inflammatory cytokines that are associated with oxidative stress mediated by the different mycotoxins (adapted from [112]) are summarized in Table 1.

### 3.4. Diet-Mediated Oxidative Stress

The supplementation of poultry diets with oils that are high in PUFAs is common as an efficient source of energy and as a means to increase palatability, to improve pellet quality, and to enhance the absorption of fat-soluble vitamins [113,114]. As mentioned earlier, PUFAs have a faster oxidation rate than saturated fats, meaning that they will become rancid more quickly. This is due to the oxidation of the reactive double bonds, which allows molecular oxygen to react with these moieties [115]. A number of additional factors, such as light exposure, the presence of catalytic transition metal ions, and high temperature during feed pelleting and storage, can lead to the production of free radicals, which in turn lead to lipid autoxidation [116,117]. The oxidation of lipids results in the production of more reactive substances, which exhibit potentially biological harmful effects and give the product an undesirable odor [118,119,120,121]. Notably, even mild oxidation can produce biologically reactive and toxic oxidation products. Lipid peroxidation results in a variety of degradation products, such as peroxides, aldehydes, and polar compounds that are differentially absorbed and metabolized. Peroxidation varies depending on the temperature, the duration of the thermal processing steps, and the composition of the oil. In this regard, feeding poultry with peroxidized oils that contain inadequate supplies of endogenous antioxidants may lead to in vivo metabolic oxidative stress [122,123,124,125]. As a result of this oxidative stress, ROS and free radical products cannot be converted into less reactive species by antioxidants and antioxidant enzymes, resulting in tissue-damaging free radicals that bind to lipids, proteins, and DNA [126] (see above). Indeed, it was demonstrated that, during the consumption of oxidized oils, reactive aldehydes accumulate in the stomach, which are adsorbed into the small intestine, where they are concentrated and metabolized in the liver [127]. Broilers that received oxidized oils had a slower growth rate, and the animals’ plasma and tissues had higher thiobarbituric acid reactive substances (TBARS) as a marker of lipid damage and a low quantity of antioxidants [128].

## 4. Anti-Inflammatory Plants and Their Active Components

PFAs can prevent chronic stress-related disorders in animals, and therefore help in improving their growth performance, by reducing their total blood cholesterol, and also by inhibiting *C. perfringens* and *E. coli* proliferation in small and large intestines [129,130]. However, there is no “magic bullet” for achieving these goals. Instead, several nutraceuticals are currently used as “alternatives antibiotics” to improve performances and gut health in animal farming [131]. Especially for commercial poultry, nutraceuticals such as phytochemicals showed promising effects, improving the intestinal microbial balance, metabolism, and the integrity of the gut due to their antioxidant, anti-inflammatory, immune modulating, and bactericidal properties [18]. In this section, we discuss polyphenols and PFAs that serve as a major source of natural antioxidants and/or anti-inflammatory compounds in poultry.

### 4.1. Polyphenols

**Figure 5:**
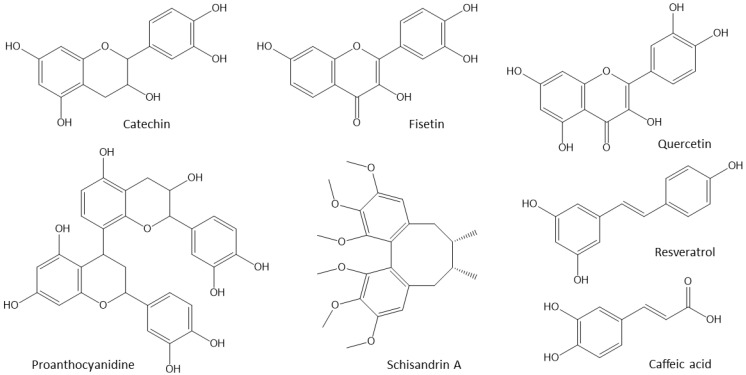
Chemical structures of some phytogenic polyphenols.

The compound family of polyphenols can be classified into four types, namely flavonoids, stilbenes, lignans, and phenolic acids. They are found in different parts of many plants (leaves, bark, stems, roots, fruits, and flowers). The chemical structures of the most common natural polyphenols are shown in Figure 5.

The antioxidant activities of polyphenols were demonstrated by various in vitro studies (Table 2). Polyphenols act directly by scavenging free radicals or indirectly through the activation of the synthesis of ROS-removing enzymes. Specifically, polyphenols scavenge free radicals via several mechanisms, including the following: (i) H-atom transfer from the OH group(s) of polyphenols to the free radical(s); (ii) single electron transfer to the free radicals [132,133,134]. It was reported that polyphenols can eliminate several ROS and RNS, such as HO^•^, ROO^•^, O2^•−^, and ONOO^−^, by these two mechanisms [135]. (iii) The final mechanism is the chelation of transition metal ions, particularly Fe^2+^ and Cu^2+^, to limit the formation of HO^•^. Polyphenols in copper/hydrogen peroxide systems exert pro-oxidant properties and prevent the formation of HO^•^ [136].

**Figure 6:**
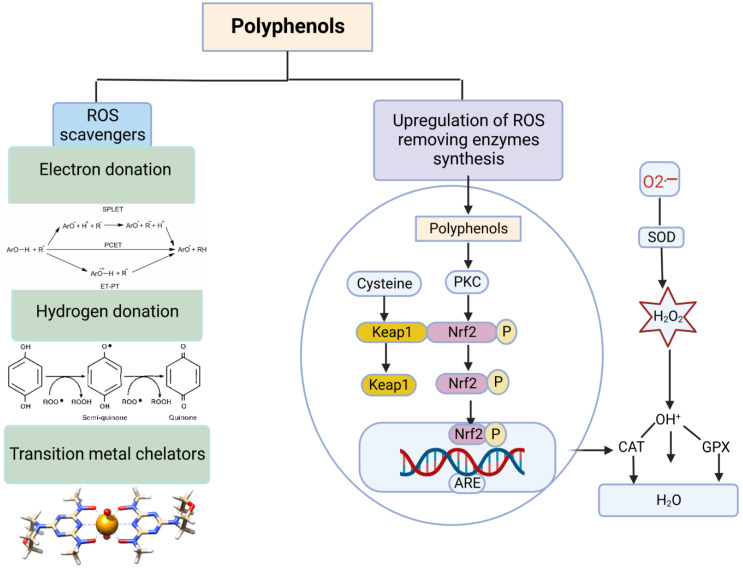
Antioxidant effect of polyphenols as natural antioxidants. Polyphenols act as antioxidants by the following two pathways: (1) scavenging of free radicals (direct) by (i) the transfer of H-atom(s) from the OH group(s) of polyphenols to the free radical(s), (ii) transfer of single electrons to the free radicals, and (iii) chelation of transition metal ions, particularly Fe^2+^ and Cu^2+^, to limit the formation of HO^•^. (2) Activation of the synthesis of ROS-removing enzymes by activation of Nrf2, which in turn translocates to the nucleus, binds to the antioxidant response elements (AREs), and thus stimulates antioxidant enzymes, including superoxide dismutase (SOD), catalase (CAT), and glutathione peroxidase (GPx). This figure was created by Biorender, modified by [153].

Polyphenols can also suppress oxidative stress by inducing antioxidant enzymes and modifying signal transduction pathways to elicit cytoprotective responses, which result in the improvement of the apparent performances, productivity, and internal physiological changes in animals [42], as shown in Figure 6. Several polyphenols activate Nrf2, which in turn stimulates the expression of antioxidant enzymes. Curcumin, for example, increases the expression of GSH-linked detoxifying enzymes, such as GSTs, GPx, and γ-GCS [137]. The green tea compound epigallocatechin-3-gallate (EGCG) is involved in the protection of neurons against oxidative stress by the activation of heme oxygenase expression [138]. Additionally, polyphenols inhibit prooxidant enzymes such as xanthine oxidase, protein kinase C and membrane-associated β-nicotinamide adenine dinucleotide (NAD(P)H) oxidase [139]. Polyphenols also alleviate NO-mediated oxidative stress [140] and prevent the oxidation of some antioxidants, such as ascorbate and tocopherols [141,142,143].

As another example, quercetin, a flavonoid compound widely present in vegetables and fruits, is well-known for its potent antioxidant effects [144]. In animals, quercetin showed anti-depressant-like actions as a result of its antioxidant, anti-inflammatory, and neuroprotective effects. The suggested mechanism of this anti-depressive effect is the modulation of neurotransmitter levels, neurogenesis, and neuronal plasticity via the stimulation of brain-derived neurotrophic factor tropomyosin receptor kinase B (BNDF/TrkB) signaling. Moreover, quercetin combats depressive-like behaviors by attenuating inflammatory responses, enhancing the expression of antioxidant enzymes, and, thus, decreasing markers of oxidative stress [145]. Additionally, silymarin from the milk thistle *Silybum marianum* contains a mixture of flavonolignans with strong antioxidant, anti-inflammatory and anticarcinogenic properties. Indeed, silymarin was shown to alleviate zeralenone-induced hepatotoxicity and reproductive toxicity in rats [146].

**Table 2:** Polyphenols as antioxidants in poultry.

Antioxidant	Dose	Main Findings	Reference
Cinnamon bark essential oil	Commercial broilers supplemented with 300 mg/kg	- Improvement of the immunological response in broiler chicks by lowering cecal *E. coli* and *Clostridium* spp. counts - Increase in the height of intestinal villi - Increase in the superoxide dismutase activity in serum	[147]
Condensed tannins from grape seed extract	Commercial broilers supplemented with 125, 250, 500, 1000 and 2000 mg/kg for 42 days	- The doses of 125 to 250 mg/kg are the optimal doses - No effects on growth performance or mortality - Decrease in the malondialdehyde content in muscle tissue - Increase in the glutathione levels in liver tissues - Decrease in the serum cholesterol and low-density lipoprotein levels	[148]
*Eucalytus* leaves extract	Layers supplemented with 0.8 g/kg. Birds suffered from acute ethanol-induced oxidative damage conditions	- Increase in glutathione peroxidase, superoxide dismutase, and total antioxidant capacity - Reduction in oxidative stress and protection of hepatic tissue	[149]
Resveratrol from *Polygonum cuspidatum*	Heat-stressed broilers supplemented 350 and 500 mg/kg for seven days (from 28 to 42 days old)	- Improvement of the average daily gain - Reduction in corticosterone, adrenocorticotropic hormone, cholesterol, triglycerides, uric acid, malonaldehyde, aspartate aminotransferase, alanine aminotransferase, and lactate dehydrogenase levels in serum - Increase in triiodothyronine, glutathione, alkaline phosphatase, total superoxide dismutase, catalase, and glutathione reductase levels in serum	[150]
Resveratrol	Heat-stressed commercial broiler supplemented with 0.2, 0.4 and 0.6 g/kg	- Increase in broiler performance - Increase in growth hormones	[151]
*Salix* spp.	Heat-stressed commercial broilers supplemented with 0.025% and 0.05% in their diet	- Reduction in serum cholesterol, triglycerides, alanine transaminase and malondialdehyde - Modulation of gastrointestinal microbiota (increase in lactobacilli)	[152]
Turmeric rhizome extract	Commercial broilers supplemented with 0.1–0.3 g/kg	- Reduction in malondialdehyde - Enhancement of the antioxidant enzyme activity - No significant alteration in serum creatinine, total proteins, or liver enzymes	[153]
Grape Proanthocyanidins	Commercial broilers supplemented with 7.5, 15 and 30 mg/kg for 42 days	- Improvement of animal performance, carcass traits, jejunum morphology and the antioxidant status (increase in superoxide dismutase and decrease in lipid peroxidation) by doses of 7.5 and 15 mg/kg of proanthocyanidins	[154]

### 4.2. Triterpenes

**Figure 7:**
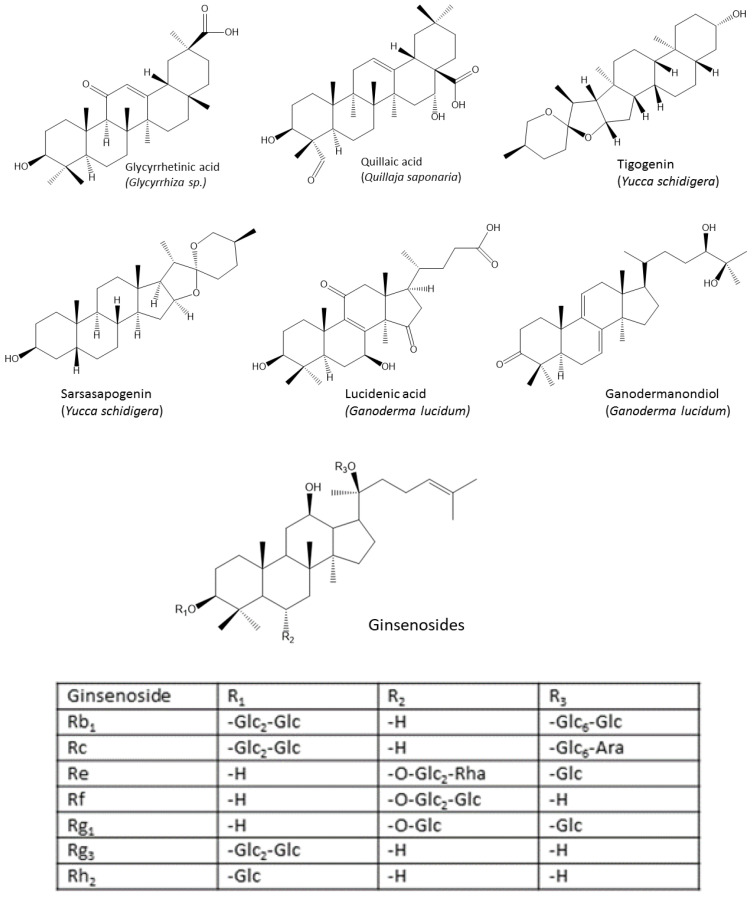
Chemical structures of triterpenes and triterpene saponins (ginsenoides).

**Figure 8:**
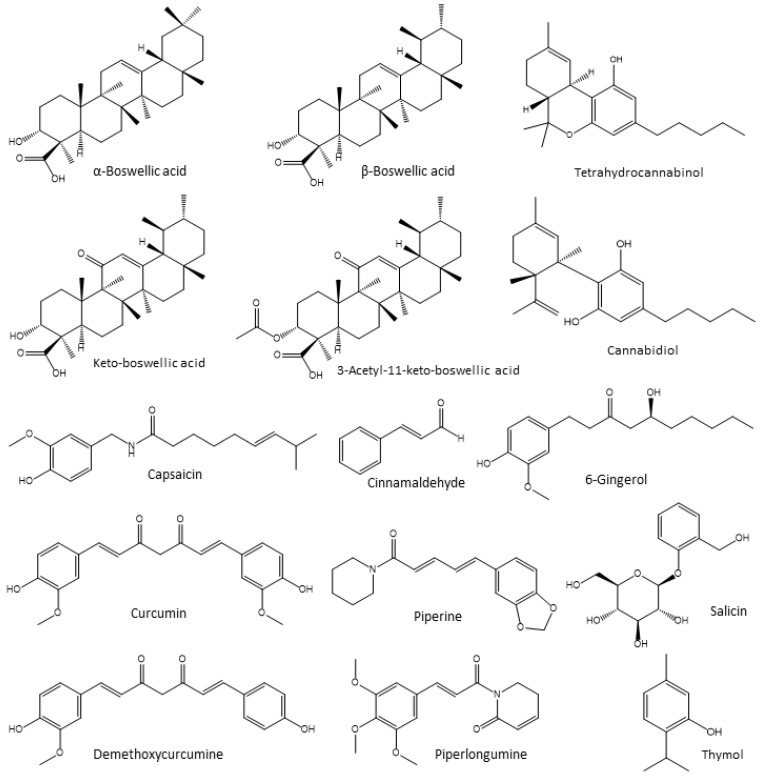
Chemical structure of other phytogenic substances with anti-inflammatory effects.

Triterpenes constitute a large group of secondary metabolites in medicinal plants and show anti-inflammatory, antiviral, antimicrobial and anti-tumor activities. They have multiple immune modulatory effects. Some chemical structures of triterpenes with anti-inflammatory effects are shown in Figure 7 and Figure 8.

As an example, glycyrrhetinic acid was shown to enhance antibody titers in chickens after vaccination against Newcastle disease [155], and steroidal saponins from *Quillaja saponaria* and *Yucca schidigera* showed anticoccidial effects in broiler chickens at a dose of 250 mg/kg [156]. *Ganoderma* triterpenoids at a dose of 10 mg/kg were able to reduce the tissue’s inflammatory status in chickens, exhibiting protective effects on the liver of the animals exposed to cadmium (140 mg/kg) [157].

Extracts of *Panax ginseng* that contained ginsenosides (triterpene saponins) were shown to ameliorate the adverse effects of heat stress by improving the intestinal barrier integrity in broilers, possibly by the upregulation of genes that encode tight junction proteins at a dose of 90 mg ginsenosides/kg feed [158]. The supplementation of chickens with ginseng improved the animal performance parameters, immunity, and meat quality [159,160,161,162]. The supplementation of chicken feed with ginseng prong powder at a dose of 0.1% or 0.2% significantly inhibited MDA in chicken breast and leg meat [159]. Additionally, the dietary supplementation of broilers with 3% of ginseng marc considerably decreased mortality and blood cholesterol levels and enhanced their carcass traits [154,160]. Similarly, a dose of 500 mg/kg of feed of dandelion (*Taraxacum*), rich in the triterpene taraxasterol and its derivatives, was reported to improve broiler performance by enhancing the tight junction and intestinal microbiota [163]. The dietary supplementation of 225 mg/kg of red ginseng root powder improved immune organ weight and increased hemoglobin and leukocytes in broilers [164].

### 4.3. Anti-Inflammatory and Antioxidant Phytogenic Feed Additives Used in Poultry

#### 4.3.1. Boswellia Extracts

Boswellia trees (family *Burseraceae*) produce frankincense oil. The resin contains volatile oils (3–8%) and triterpenes (30–60%), especially α- and β-boswellic acids, 11-keto-boswellic acid (KBA), and 3-acetyl-11-keto-boswellic acid (AKBA) [165] (Figure 8). Boswellia has an anti-inflammatory impact on the suppression of 5-lipoxygenase (5-LOX), lowering cytokine levels (Ils and TNF-α), and decreasing ROS production. *B. serrata* (0.5, 1 and 1.5 g BS/kg diet) was shown to improve the antioxidant status, boost the globulin levels and SOD, and stimulate the secretion of digestive enzymes (amylase and lipase), while decreasing total cholesterol, LDL, and MDA in broilers [166]. The addition of *Boswellia* (3% and 4%) to broiler chicken diets enhanced the body weight, digestion efficiency, and carcass traits of the chickens [167]. *B. serrata* (containing 24% boswellic acids) and *Salix alba* (containing 43% of salicin) at a dose of 0.3% in poultry feed for 12 weeks caused considerably greater antibody titers against the infectious bronchitis virus in Leghorn chickens. There were no variations in their performance metrics, blood analytes, or IgA levels. However, a depressive effect, a drop in egg mass, and an increase in water intake were observed [168].

#### 4.3.2. Cannabis

*Cannabis sativa* L., *C. indica* Lam. and *C. ruderalis* Janisch belong to the *Cannabaceae* family of dioecious flowering plants. Due to the cannabinoids included in cannabis, such as the psychoactive tetrahydrocannabinol (THC) and the non-psychoactive cannabidiol (CBD) (Figure 8), it has been used for decades as an analgesic, antispasmodic, and anti-inflammatory drug (CBD) [169]. Phytocannabinoids are synthesized in the glandular trichomes of the female *Cannabis* blooms [170]. These bioactive substances bind to the receptors CB1 (primarily released in the brain), and CB2 (found mainly on immune cells). It was shown that cannabis functions as an anti-inflammatory agent by upregulating T-regulatory cells and downregulating cytokine and chemokine release [171]. Additionally, exogenous cannabinoids have the potential as therapeutic agents for a variety of inflammatory disorders. In poultry, the *Cannabis* seed (0.3% in feed) alone or in combination with dill (0.3% in feed) was found to promote the intestinal health and serum quality of commercial broiler chickens [172]. This combination significantly reduced both AST and ALT concentrations; however, the alkaline phosphatase concentrations were not affected. CBD alone also showed beneficial effects in animal breeding [169] in chickens at a dose of 15 g/kg. It was also found that *C. sativa* extract in combination with nano-selenium improved gut integrity and influences the response to *Clostridia* infection [169].

#### 4.3.3. Capsaicin

The primary capsaicinoid in chili pepper is capsaicin, a naturally occurring bioactive compound (Figure 8). It has attracted considerable scientific interest for its multiple pharmacological and biological functions, such as its ability to serve as an antioxidant [173] and anti-inflammatory substance [174]. The phenolic hydroxyl group of capsaicin can effectively lower the activity of free radicals by transferring hydrogen [175]. Moreover, the phenolic hydroxy group prevents the production of free radicals that require metal ions [176]. The anti-inflammatory activities of capsaicin may be explained by its modulation of pro-inflammatory mediators [177]. In rats suffering from gastritis induced by acetylsalicylic acid, capsaicin reduced the expression of genes that encode for TNF-α, IL-1β, and IL-6, resulting in a decrease in the infiltration of inflammatory cells [178]. The tendency of capsaicin to substantially diminish the release of COX-2 mRNA is thought to be the reason for its anti-inflammatory effects [179].

Additionally, studies have shown that capsaicin suppresses free radical-induced oxidative DNA damage, lipid peroxidation, and oxidative degradation pathways [179,180]. The dietary supplementation of capsaicin at a dose of 150 mg/kg stimulated the appetite of laying ducks, leading to increased feed intake and an improvement in egg production performance [181]. The authors suggested that these positive effects of capsaicin could be attributed to the activation of the calcium signaling pathway and the antioxidant effects. According to Liu et al., supplementing diets with 80 mg/kg of natural capsaicin extract could enhance broiler growth performance, nutrient digestibility, antioxidant status, immunological functions, and carcass traits [179].

#### 4.3.4. Cinnamaldehyde

Cinnamaldehyde (Figure 8) is the principal bioactive component in cinnamon, which belongs to the family *Lauraceae* (rowan family). There are only a few species that are economically important worldwide and these include *Cinnamomum zeylanicum*, *C. cassia*, *C. burmanni* and *C. loureiori* [182,183]. The ingredients of cinnamon extract, such as alkaloids, coumarins, curcuminoids, flavonoids, phenols, tannins, terpenoids, volatiles, and xanthones, are well-known for their biological effects, including their antioxidative, antimicrobial, and anti-inflammatory properties [182]. Cinnamaldehyde has been shown to decrease the expression of several cytokines, such as IL-1 β, IL-6, and TNF-α, as well as iNOS and COX-2, in in vitro studies [184]. Moreover, it stimulated the secretion of IL-10 in LPS-activated murine macrophage-like cells (J774A.1).

Several in vivo studies confirmed that cinnamaldehyde has anti-inflammatory effects that, for example, resulted in improved poultry immunity in terms of antigen presentation, and humoral cellular immune responses [185]. Cinnamaldehyde dosages of 1.2 to 5.0 g/mL activated macrophages to release larger quantities of NO, while a dose of 0.6 to 2.5 g/mL inhibited chicken tumor cell proliferation. A dose of 10 and 100 µg/mL of cinnamaldehyde exhibited anticoccidial effects against *E. tenella* [186]. In contrast, a cinnamaldehyde dose of 14.4 mg/kg boosted the expression of pro-inflammatory cytokines IL-1, IL-6, IL-15, and IFN-γ in vivo. Moreover, it was found that cinnamaldehyde improved the body weight gain of chickens infected with *E. acervulina* or *E. maxima* [186]. More studies are needed to determine whether cinnamaldehyde has an immunoregulatory effect in a dose-dependent manner.

#### 4.3.5. Curcumin

*Curcuma longa* (turmeric), which belongs to the family *Zingiberaceae*, is widely used as a spice, food preservative and coloring agent, and for medicinal applications [187]. Since the 19th century, various *Curcuma* species have been employed in medicine. The *Curcuma* rhizome has an intense yellow color and contains curcumin (70% diferuloylmethane), 15% demethoxycurcumin, and 3% bis-dimethoxycurmarin [188] (Figure 8). Curcumin was found to have antioxidant [189] and anti-infective activities, lowering the severity of necrotic enteritis [190], salmonellosis [190,191], aflatoxicosis [192], and coccidiosis [193]. Indeed, *Curcuma* is one of the strongest natural antioxidants with anti-inflammatory, antiviral, antimicrobial, cleansing, anticancer, antioxidant, antiseptic, radioprotective, and cardioprotective effects. It promotes pancreatic and liver functions and has a cleansing impact on the blood [188]. In chicken macrophages, turmeric extract enhanced the expression of IL-1, IL-6, IL-12, IL-18, and TNF superfamily 15 [194]. Several studies were also carried out to estimate the impact of curcuminoids on the immune response of swine [195,196,197,198]. Curcuminoid supplementation markedly decreased the mRNA expression patterns of IL-1β, mucin 2, COX-2, and p38 MAPK in ileal mucosa [196] and serum TNF-α concentration [197]. In conclusion, curcumin predominantly alters the p38 MAPK pathway, and thus suppresses the downstream formation of IL-1β, IL-6, and TNF-α. In broilers, curcumin at a dose of 1000 and 2000 mg/kg feed decreased the lipid profile in the liver and plasma and altered the expression of genes involved in lipogenesis and lipolysis, including acetyl CoA carboxylase, fatty acid synthase, sterol regulatory element-binding protein 1C, ATP-citrate lyase, peroxisome proliferator-activated receptor-α, and carnitine palmitoyl transferase-I [199]. Yadav and co-workers found that the antioxidant activities, lesion severity, and shedding of oocysts in commercial broilers were positively affected by curcumin at a dose of 200 mg/kg feed. It was also suggested that curcumin alone or in combination with other bioactive substances could enhance intestinal health in commercial broilers [198].

#### 4.3.6. Ginger Extracts

Ginger rhizome (*Zingiber officinale* Roscoe, *Zingiberaceae*) is believed to be native to the Indian subcontinent and other regions of Southern Asia. It is a valuable plant with numerous ethnomedicinal and nutritional properties, and it is frequently employed all over the world as a spice, flavoring, and herbal remedy [200]. Ginger is rich in many bioactive substances, including phenolics and terpenes. The phenolic substances, primarily gingerols (Figure 8), shogaols, and paradols, are responsible for numerous bioactivities [201]. Indeed, various reports suggest that ginger and its compounds have antioxidant [202], anti-inflammatory [203], antibacterial [204], and anticancer [205,206,207] properties. In poultry farming, dietary supplementation of ginger powder at a dose of 10 or 20 g/kg feed exhibited an antioxidant effect by increasing SOD, GSH-PX, and the total antioxidant capacity (T-AOC) but decreased the MDA levels in serum. It also increased the SOD and decreased MDA levels in the egg yolk in a dose-dependent manner [208]. The antioxidant components such as gingerols, shogaols, gingerdiols, gingerdiones and some related phenolic ketone derivatives are probably responsible for the improved antioxidant status of ginger powder supplementation [208,209,210]. Ginger inhibited lipid peroxidation by enhancing the oxidative processes [208].

The ginger extract obtained from *Zingiber officinale* and *Alpinia galanga* inhibited the expression of numerous genes associated with the inflammatory processes [200]. It reduced prostaglandin synthesis [211] by inhibiting COX-1 and COX-2. Additionally, it also blocks leukotriene synthesis by suppressing 5-LOX [200]. More recently, it was established that ginger extract enhances the immune system, and boosts the antioxidant and anti-inflammatory capacities of layers [211].

#### 4.3.7. Piperamides

Black pepper (*Piper nigrum*), which belongs to the family *Piperaceae*, is rich in GPx and glucose-6-phosphate dehydrogenase [212]. Black pepper is employed in Eastern medicine for the treatment of pain symptoms and infections. The piperine analogue piperlongumine (Figure 8) has antioxidant properties [213], and can enhance the uptake of selenium, vitamin B complex, beta-carotene, and curcumin [188,214]. Abou-Elkhair et al. found that dietary supplements with 0.5% black pepper improved the animal performance and health status of commercial broilers. It has a strong action against free radicals and influences benzopyrene metabolism through cytochrome P_450_, which is crucial for the metabolism and transportation of xenobiotics [215]. The compound promotes the thermogenesis of lipids [216] and increases the flow of digestive juice [217]. It helps in maintaining the circulatory system of the liver and provides protection against DNA damage. Piperine also showed some benefits regarding the ultrastructure of intestinal microvilli and gut motility, which improved the absorption of micronutrients.

It is also interesting to note that the guineensin extract obtained from black pepper has anti-inflammatory activity, inhibiting the uptake of endocannabinoids by the cells. Reynoso-Moreno et al. [218] assessed the effects of guineensin on endotoxemia and acute inflammation in mice models. The strong pharmacological action of guineinin may also add to the anti-inflammatory effects of black pepper [188,218].

It was shown that piperlongumine is effective against LPS-induced disrupted endothelial barriers in cell and animal models [219]. It also suppressed IL-6 and TNF-α by inhibiting the stimulation of NF-κB and extracellular signal-regulated kinase (ERK). In prostate cancer cells, piperlongumine showed anticancer action, including the suppression of NF-B activity [220], which in turn diminished the reduction in trafficking of p50 and p65.

In broilers, black pepper supplementation improved their body weight. However, it did not affect the feed intake, carcass yield, or relative weights of internal organs, including the liver, gizzard, proventriculus, heart, spleen, thymus, and bursa of Fabricius. In addition, the serum parameters (total protein, albumin, globulin, glucose, cholesterol, triglyceride, and liver enzymes) did not exhibit a significant effect [221,222,223]. In another study, it was found that body weight gain was not influenced by black pepper supplementation [224]. In contrast, Al-Kassie and co-workers found that the supplementation of broilers with a mixture of *Piper nigrum* and *Capsicum annum* black pepper improved the animal performance and reduced their blood cholesterol level [225].

#### 4.3.8. Salix Extracts

The bark and leaves of willows (genus: Salix, family: *Salicaceae*) contain salicin (Figure 8) and its derivatives, including polyphenols, and flavonoids. The biological activities of Salix extracts, including its antioxidant, anti-inflammatory, analgesic, and antipyretic properties, have been repeatedly documented [226]. The underlying mechanism involves the suppression of TNF-α, IL-1ß, IL-6, cyclooygenase-1 (COX-1), and COX-2 expression. The efficacy of Salix has also been studied in poultry. *Salix babylonica* extract improved animal performance and the heat tolerance of broilers kept under constant heat stress (35 °C) [227].

In commercial broilers, *Salix* L. bark powder at a dose of 0.05% in their diet exhibited a lower MDA, GSH, and lipid peroxidation indicator (thiobarbituric acid reactive substances) in the liver tissues. However, no significant effect of hepatic SOD activity was found. Moreover, *Salix* L. bark modulated the gut microbiota by increasing *Lactobacilli*, and decreasing *E. coli* and *staphylococci* [228]. *S. alba* bark extract (1% of diet) induced hypocholesterolemia and reduced the proliferation of pathogenic bacteria (*Enterobacteriaceae*, *E. coli* and *Staphylococci*) in the caecum, but did not show significant differences of the growth performance in broilers [229].

#### 4.3.9. Thyme

Multiple studies have documented the properties of thyme and its essential oils, particularly the monoterpenes, thymol and carvacrol (Figure 8), against a variety of disorders. Thymol and carvacrol possess multi-pharmacological capabilities, including antioxidant and anti-inflammatory properties. Thyme supplementation at a dose of 2% reduced the levels of cholesterol, total saturated fatty acids, and MDA, while it increased ω−3 fatty acid contents in egg yolk. However, it reduced the serum cholesterol and triglyceride levels and increased antibody titers against sheep red blood cells (SRBC) [230]. Thyme oil reduced the synthesis and gene expression of TNF-α, IL-1B, and IL-6 in activated macrophages in a dose-dependent manner, with upregulation of IL-10 secretion [231].

Additionally, it inhibited dendritic cell maturation and stimulation of T cell proliferation in vitro [232]. Among other pathways, thymol was found to inhibit the phosphorylation of NF-κB and MAPKs, and downregulated IL-6, TNF-α, iNOS and COX-2 in LPS-stimulated murine mammary epithelial cells [233]. Thymol at a dose of 10, 20, and 40 μg/mL also prevented the activation of the MAPKs I-B, NF-B p65, ERK, JNK, and p38 in mouse mammary epithelial cells in a dose-dependent manner that had been activated by LPS [233,234]. The anti-inflammatory properties of thyme suggest that it is suitable for use in animal production, as shown in the previous studies. In poultry, it has been demonstrated that thyme oil at a dose of 100 mg/kg also promotes the secretion of digestive enzymes, which enhances nutrient digestion [235]. However, no significant effects on the growth performance were observed. Supplementation of the diets with 5 g/kg of thyme oil reduced the pro-inflammatory mediators and improved the immune system and animal performance of broilers [234].

## 5. Challenges and Future Prospects

Phytogenic compounds have been evaluated as potential alternatives to antimicrobials in poultry [88]. However, the bioavailability, rate of absorption, and cost-effective delivery methods for phytogenic compounds make their feasibility and application on a commercial scale complicated [236]. The efficacy of phytogenic compounds has not been regulated since most of these compounds are generally accepted as safe by the US Food and Drug Administration. As a result, the efficaciousness of polyphenolic compounds derived from the same origin and manufacturers may vary. For example, the stoichiometry and stability of flavonoids depend on several factors, including the plant origin and quality, as well on the method of extraction [237]. Additionally, antioxidants that bear only one hydroxyl group, such as ferulic acids, do not chelate metals [238]. Although the biochemical structure of polyphenols results in their high biological activity as antioxidants in vitro, their biological efficiency in animals is hindered, due to the poor oral bioavailability of the polyphenols, which is explained by the contradictory findings from both in vivo and in vitro experiments [239,240]. The lower efficiency of polyphenols in vivo could be attributed to the following several factors [236]: (i) the low uptake and assimilation of polyphenolic substances may lead to insufficient minimal concentrations in target tissues, meaning they are not effective as scavengers of free radicals; (ii) the extensive biotransformation that occurs in the liver and intestinal tract may influence the functional forms of these substances, which in turn adversely impacts biological activities, including the antioxidant properties [142], and/or (iii) they may be metabolized and quickly eliminated from the bloodstream [241]. For these reasons, the observed in vivo antioxidant effects could be indirect effects that occur through the upregulation of antioxidant defenses by the substantial protective effect in the gastrointestinal tract and their effects on Nrf2 and NF-κB [140]. Therefore, methods to improve the bioavailability and absorption of polyphenolic compounds, but also of other phytogenic compounds, urgently require further investigation.

Dietary inclusion of unprotected natural compounds, in particular polyphenolic compounds, is not cost-effective, since most phytogenic compounds are degraded in the upper small intestine [242]. Mainly driven by this fact, microencapsulation is a promising method to protect bioactive phytogenic substances from oxidation, and degradation during storage, and to increase their bioavailability in piglets [243]. This method also reduces the early degradation of the compound in the small intestine, and thus ensures its delivery to the lower intestinal tract. For microencapsulation, the following two main carriers have been described: polymer-based particles and lipid-based particles. Polymer-based particles, such as polysaccharide protein scaffolds, are stable both thermally and mechanically. They are also characterized by their nutritional value, affordability, and ease of production. Nevertheless, there are still some limitations due to their low loading capacity, low encapsulation efficiency, and release into the gastrointestinal tract [244]. An alternative method using alginate–whey protein as a carrier to increase the delivery of carvacrol in the chicken intestine has been evaluated [245]. Compared to the administration of unprotected carvacrol, the alginate–whey protein microparticles increased the amount of monoterpene in the ileum by 17%.

Lipid-based particles demonstrate considerable encapsulation efficiency, loading capacity, and releasing ability in the gastrointestinal tract. Examples of these particles include liposomes and vegetable oils. Their low mechanical and thermal stabilities, however, are a drawback. Liposomes cannot be used for mass production due to their high costs, challenging preparation procedures, and constrained capacity [246].

Another factor that impacts the bioavailability and absorption of phytogenic substances that must be considered is the intestinal microbiota. Lactic acid bacteria derived from chicken cecal contents have been shown to increase the bioavailability of flavonoids by increasing flavonoid hydrolysis, but this was affected by the carbon source available for microbial fermentation [247]. Optimizing the method of encapsulation and understanding the impacts of microbial fermentation on the rate of degradation and kinetics of phytogenic compounds are also required [236].

Taken together, the variability due to the volatility of many phytogenic compounds, the method of encapsulation, and the factors within the hosts need to be considered when further evaluating these compounds. The evaluation of the antioxidant activities of different bioactive substances, especially when evaluating the synergistic effects of multiple compounds, is of major interest.

## Figures and Tables

**Table 1:** Modulatory effect of mycotoxins on intracellular antioxidants and pro-inflammatory cytokines.

Mycotoxin	Downregulation of Intracellular Antioxidants	Upregulation of Pro-Inflammatory Cytokines
AFB1	Nrf2, CAT, GPx; SOD	Cytokines, NO; NO_2_
DON	CAT, GPx; SOD	AP-1; ERK-MAPK
OTA	Nrf2, CAT, GPx; SOD	Fenton reaction
ZEN	CAT, GPx; SOD	CoX-2, cytokines; iNOS
T-2	Nrf2, CAT, GPx, GPx; SOD	Cytokines, iNOS; NO

AFB1, aflatoxin B1; DON, deoxynivalenol; NIV, nivalenol; FB1, fumonisin B1; OTA, ochratoxin A; ZEN, zearalenon. Nrf2, erythroid 2-related factor 2; CAT, catalase; GPx, glutathione peroxidase; SOD, superoxide dismutase; NO, nitric oxide; NO_2_, nitrogen dioxide; AP-1, activator protein 1; ERK-MAPK, extracellular signal-regulated kinase-mitogen-activated protein kinase; CoX-2, cyclooygenase-2; iNOS, inducible nitric oxide synthetase.

## Data Availability

Not applicable.

## References

[1] Quintana-Cabrera R., Mehrotra A., Rigoni G., Soriano M.E. (2018). Who and how in the regulation of mitochondrial cristae shape and function. Biochem. Biophys. Res. Commun..

[2] Mannella C.A. (2006). The relevance of mitochondrial membrane topology to mitochondrial function. Biochim. Et Biophys. Acta (BBA)-Mol. Basis Dis..

[3] Papadopoulos V., Miller W.L. (2012). Role of mitochondria in steroidogenesis. Best Pract. Res. Clin. Endocrinol. Metab..

[4] Osellame L.D., Blacker T.S., Duchen M.R. (2012). Cellular and molecular mechanisms of mitochondrial function. Best Pract. Res. Clin. Endocrinol. Metab..

[5] Maechler P. (2012). Mitochondrial signal transduction in pancreatic β-cells. Best Pract. Res. Clin. Endocrinol. Metab..

[6] Fransen M., Nordgren M., Wang B., Apanasets O. (2012). Role of peroxisomes in ROS/RNS-Imetabolism: Implications for human disease. Biochim. Biophys. Acta.

[7] Li J.-M., Shah A.M. (2004). Endothelial cell superoxide generation: Regulation and relevance for cardiovascular pathophysiology. Am. J. Physiol. Regul. Integr. Comp. Physiol..

[8] Zorov D.B., Juhaszova M., Sollott S.J. (2014). Mitochondrial reactive oxygen species (ROS) and ROS-induced ROS release. Physiol. Rev..

[9] Nowak J.Z. (2013). Oxidative Stress, Polyunsaturated Fatty acids-derived oxidation products and bisretinoids as potential inducers of CNS diseases: Focus on age-related macular degeneration. Pharmacol. Rep..

[10] Gaschler M.M., Stockwell B.R. (2017). Lipid peroxidation in cell death. Biochem. Biophys. Res. Commun..

[11] Kiecolt-Glaser J.K. (2010). Stress, food, and inflammation: Psychoneuroimmunology and nutrition at the cutting edge. Psychosom. Med..

[12] Reuter S., Gupta S.C., Chaturvedi M.M., Aggarwal B.B. (2010). Oxidative stress, inflammation, and cancer: How are they linked?. Free Radic. Biol. Med..

[13] Hussain T., Tan B., Yin Y., Blachier F., Tossou M.C.B., Rahu N. (2016). Oxidative stress and inflammation: What polyphenols can do for us?. Oxid. Med. Cell. Longev..

[14] Mahesh G., Anil Kumar K., Reddanna P. (2021). Overview on the discovery and development of anti-inflammatory drugs: Should the focus be on synthesis or degradation of PGE2?. J. Inflamm. Res..

[15] Windisch W., Schedle K., Plitzner C., Kroismayr A. (2008). Use of phytogenic products as feed additives for swine and poultry. J. Anim. Sci..

[16] Mahfuz S., Shang Q., Piao X. (2021). Phenolic Compounds as natural feed additives in poultry and swine diets: A review. J. Anim. Sci. Biotechnol..

[17] Achilonu M.C., Umesiobi D.O. (2015). Bioactive phytochemicals: Bioactivity, sources, preparations, and/or modifications via silver tetrafluoroborate mediation. J. Chem..

[18] Estévez M. (2015). Oxidative damage to poultry: From farm to fork. Poult. Sci..

[19] Ferro E., Goitre L., Retta S.F., Trabalzini L. (2012). The Interplay between ROS and Ras GTPases: Physiological and pathological implications. J. Signal. Transduct..

[20] Pizzino G., Irrera N., Cucinotta M., Pallio G., Mannino F., Arcoraci V., Squadrito F., Altavilla D., Bitto A. (2017). Oxidative stress: Harms and benefits for human health. Oxid. Med. Cell. Longev..

[21] Nita M., Grzybowski A. (2016). The role of the reactive oxygen species and oxidative stress in the pathomechanism of the age-related ocular diseases and other pathologies of the anterior and posterior eye segments in adults. Oxid. Med. Cell. Longev..

[22] Costa M., Sezgin-Bayindir Z., Losada-Barreiro S., Paiva-Martins F., Saso L., Bravo-Díaz C. (2021). Polyphenols as antioxidants for extending food shelf-life and in the prevention of health diseases: Encapsulation and Interfacial Phenomena. Biomedicines.

[23] Bedard K., Krause K.-H. (2007). The NOX Family of ROS-Generating NADPH Oxidases: Physiology and pathophysiology. Physiol. Rev..

[24] Brandes R.P., Weissmann N., Schröder K. (2014). Nox Family NADPH Oxidases: Molecular mechanisms of activation. Free Radic. Biol. Med..

[25] Shahidi F. (2015). Handbook of Antioxidants for Food Preservation.

[26] Halliwell B. (2007). Dietary Polyphenols: Good, bad, or indifferent for your health?. Cardiovasc. Res..

[27] Losada-Barreiro S., Bravo-Díaz C. (2017). Free Radicals and Polyphenols: The redox chemistry of neurodegenerative diseases. Eur. J. Med. Chem..

[28] Hayes J.D., Strange R.C. (1995). Potential contribution of the glutathione S-transferase supergene family to resistance to oxidative stress. Free Radic. Res..

[29] Pickett C.B., Lu A.Y. (1989). Glutathione S-transferases: Gene structure, regulation, and biological function. Annu. Rev. Biochem..

[30] Huang Y., Li W., Su Z., Kong A.-N.T. (2015). The complexity of the Nrf2 pathway: Beyond the antioxidant response. J. Nutr. Biochem..

[31] Retsky K.L., Freeman M.W., Frei B. (1993). Ascorbic acid oxidation product(s) protect human low density lipoprotein against atherogenic modification. anti- rather than prooxidant activity of vitamin c in the presence of transition metal ions. J. Biol. Chem..

[32] Miyazawa T., Burdeos G.C., Itaya M., Nakagawa K., Miyazawa T. (2019). Vitamin E: Regulatory redox interactions. IUBMB Life.

[33] Wang X., Quinn P.J. (1999). Vitamin E and its function in membranes. Prog. Lipid Res..

[34] Marí M., Morales A., Colell A., García-Ruiz C., Kaplowitz N., Fernández-Checa J.C. (2013). Mitochondrial glutathione: Features, Regulation and role in disease. Biochim. Et Biophys. Acta (BBA)-Gen. Subj..

[35] Matés J.M., Sánchez-Jiménez F. (1999). Antioxidant enzymes and their implications in pathophysiologic processes. Front. Biosci..

[36] Xiao W., Loscalzo J. (2020). Metabolic responses to reductive stress. Antioxid. Redox Signal..

[37] Quinteiro-Filho W.M., Ribeiro A., Ferraz-de-Paula V., Pinheiro M.L., Sakai M., Sá L.R.M., Ferreira A.J.P., Palermo-Neto J. (2010). Heat stress impairs performance parameters, induces intestinal injury, and decreases macrophage activity in broiler chickens. Poult. Sci..

[38] Valko M., Leibfritz D., Moncol J., Cronin M.T.D., Mazur M., Telser J. (2007). Free radicals and antioxidants in normal physiological functions and human disease. Int. J. Biochem. Cell Biol..

[39] Jin X., Liu Q., Jia L., Li M., Wang X. (2015). Pinocembrin attenuates 6-ohda-induced neuronal cell death through Nrf2/ARE pathway in SH-SY5Y cells. Cell. Mol. Neurobiol..

[40] Su L.-J., Zhang J.-H., Gomez H., Murugan R., Hong X., Xu D., Jiang F., Peng Z.-Y. (2019). Reactive oxygen species-induced lipid peroxidation in apoptosis, autophagy, and ferroptosis. Oxidative Med. Cell. Longev..

[41] Farley N., Pedraza-Alva G., Serrano-Gomez D., Nagaleekar V., Aronshtam A., Krahl T., Thornton T., Rincón M. (2006). P38 mitogen-activated protein kinase mediates the fas-induced mitochondrial death pathway in CD8+ T cells. Mol. Cell. Biol..

[42] Lee M.T., Lin W.C., Yu B., Lee T.T. (2016). Antioxidant capacity of phytochemicals and their potential effects on oxidative status in animals—A review. Asian-Australas. J. Anim. Sci..

[43] Blackwell T.S., Blackwell T.R., Holden E.P., Christman B.W., Christman J.W. (1996). In vivo antioxidant treatment suppresses nuclear factor-kappa B activation and neutrophilic lung inflammation. J. Immunol..

[44] Cuzzocrea S., Riley D.P., Caputi A.P., Salvemini D. (2001). Antioxidant therapy: A new pharmacological approach in shock, inflammation, and ischemia/reperfusion injury. Pharmacol. Rev..

[45] Laskin D.L., Sunil V.R., Gardner C.R., Laskin J.D. (2011). Macrophages and tissue injury: Agents of defense or destruction?. Annu. Rev. Pharmacol. Toxicol..

[46] Gloire G., Legrand-Poels S., Piette J. (2006). NF-KappaB activation by reactive oxygen species: Fifteen years later. Biochem. Pharmacol..

[47] Cai D., Khor S. (2019). “Hypothalamic microinflammation” paradigm in aging and metabolic diseases. Cell Metab..

[48] Zhang X., Zhang G., Zhang H., Karin M., Bai H., Cai D. (2008). Hypothalamic IKKbeta/NF-KappaB and ER stress link overnutrition to energy imbalance and obesity. Cell.

[49] Meng Q., Cai D. (2011). Defective hypothalamic autophagy directs the central pathogenesis of obesity via the IkappaB kinase Beta (IKKbeta)/NF-KappaB pathway. J. Biol. Chem..

[50] Jones S.V., Kounatidis I. (2017). Nuclear Factor-kappa B and alzheimer disease, unifying genetic and environmental risk factors from cell to humans. Front. Immunol..

[51] Poljsak B., Šuput D., Milisav I. (2013). Achieving the balance between ROS and antioxidants: When to use the synthetic antioxidants. Oxidative Med. Cell. Longev..

[52] Celi P. (2011). Biomarkers of oxidative stress in ruminant medicine. Immunopharmacol. Immunotoxicol..

[53] Montuschi P., Barnes P.J., Roberts L.J. (2004). Isoprostanes: Markers and mediators of oxidative stress. FASEB J..

[54] Dalle-Donne I., Rossi R., Giustarini D., Milzani A., Colombo R. (2003). Protein Carbonyl groups as biomarkers of oxidative stress. Clin. Chim. Acta.

[55] Collins A.R. (2014). Measuring oxidative damage to dna and its repair with the comet assay. Biochim. Biophys. Acta.

[56] Tellez-Isaias G., Eisenreich W., Shehata A.A. (2022). Nutraceuticals to mitigate the secret killers in animals. Vet. Sci..

[57] Bickler S.W., Prieto J.M., Cauvi D.M., De Cos V., Nasamran C., Ameh E., Amin S., Nicholson S., Din H., Mocumbi A.O. (2020). Differential Expression of nuclear genes encoding mitochondrial proteins from urban and rural populations in Morocco. Cell Stress Chaperones.

[58] Korniluk A., Koper O., Kemona H., Dymicka-Piekarska V. (2017). From inflammation to cancer. Ir. J. Med. Sci..

[59] Mishra B., Jha R. (2019). Oxidative stress in the poultry gut: Potential challenges and interventions. Front. Vet. Sci..

[60] Zhao H., He Y., Li S., Sun X., Wang Y., Shao Y., Hou Z., Xing M. (2017). Subchronic arsenism-induced oxidative stress and inflammation contribute to apoptosis through mitochondrial and death receptor dependent pathways in chicken immune organs. Oncotarget.

[61] Fasano A. (2020). All disease begins in the (leaky) gut: Role of zonulin-mediated gut permeability in the pathogenesis of some chronic inflammatory diseases. F1000Research.

[62] Sekirov I., Russell S.L., Antunes L.C.M., Finlay B.B. (2010). Gut microbiota in health and disease. Physiol Rev..

[63] Dimitrov D.V. (2011). The human gutome: Nutrigenomics of the host-microbiome interactions. OMICS.

[64] Fukui H., Xu X., Miwa H. (2018). Role of gut microbiota-gut hormone axis in the pathophysiology of functional gastrointestinal disorders. J. Neurogastroenterol. Motil..

[65] Megur A., Baltriukienė D., Bukelskienė V., Burokas A. (2020). The microbiota-gut-brain axis and Alzheimer’s disease: Neuroinflammation is to blame?. Nutrients.

[66] Neuman H., Debelius J.W., Knight R., Koren O. (2015). Microbial endocrinology: The interplay between the microbiota and the endocrine system. FEMS Microbiol. Rev..

[67] Maslowski K.M., Mackay C.R. (2011). Diet, gut microbiota and immune responses. Nat. Immunol..

[68] Stecher B. (2015). The Roles of inflammation, nutrient availability and the commensal microbiota in enteric pathogen infection. Microbiol. Spectr..

[69] Lara L., Rostagno M. (2013). Impact of heat stress on poultry production. Animals.

[70] Pawar S.S., Basavaraj S., Dhansing L.V., Pandurang K.N., Sahebrao K.A., Vitthal N.A., Pandit B.M., Kumar B.S., ICAR-National Institute of Abiotic Stress Management (2016). Assessing and mitigating the impact of heat stress in poultry. Adv. Anim. Vet. Sci..

[71] Hirakawa R., Nurjanah S., Furukawa K., Murai A., Kikusato M., Nochi T., Toyomizu M. (2020). Heat stress causes immune abnormalities via massive damage to effect proliferation and differentiation of lymphocytes in broiler chickens. Front. Vet. Sci..

[72] Mount L.E. (1978). Heat transfer between animal and environment. Proc. Nutr. Soc..

[73] Lasiewski R. (1969). Physiological responses to heat stress in the poorwill. Am. J. Physiol.-Leg. Content.

[74] Ortega A.D.S.V., Szabó C. (2021). Adverse effects of heat stress on the intestinal integrity and function of pigs and the mitigation capacity of dietary antioxidants: A review. Animals.

[75] Yi H., Xiong Y., Wu Q., Wang M., Liu S., Jiang Z., Wang L. (2020). Effects of Dietary supplementation with L-arginine on the intestinal barrier function in finishing pigs with heat stress. J. Anim. Physiol. Anim. Nutr..

[76] Dokladny K., Zuhl M.N., Moseley P.L. (2016). Intestinal epithelial barrier function and tight junction proteins with heat and exercise. J. Appl. Physiol..

[77] Alhenaky A., Abdelqader A., Abuajamieh M., Al-Fataftah A.-R. (2017). The effect of heat stress on intestinal integrity and *salmonella* invasion in broiler birds. J. Therm. Biol..

[78] Mercer E.H., Singh A.P. (1975). Endosymbiosis and cellular tolerance in the hawaiian soft coral sarcothelia edmondsoni verrill. Adv. Exp. Med. Biol..

[79] Pearce S.C., Mani V., Boddicker R.L., Johnson J.S., Weber T.E., Ross J.W., Rhoads R.P., Baumgard L.H., Gabler N.K. (2013). Heat Stress Reduces Intestinal barrier integrity and favors intestinal glucose transport in growing pigs. PLoS ONE.

[80] Wu Q.J., Liu N., Wu X.H., Wang G.Y., Lin L. (2018). Glutamine alleviates heat stress-induced impairment of intestinal morphology, intestinal inflammatory response, and barrier integrity in broilers. Poult. Sci..

[81] Yu Q., Tang C., Xun S., Yajima T., Takeda K., Yoshikai Y. (2006). MyD88-dependent signaling for IL-15 production plays an important role in maintenance of CD8 alpha alpha TCR alpha beta and TCR gamma delta intestinal intraepithelial lymphocytes. J. Immunol..

[82] von Meyenburg C., Hrupka B.H., Arsenijevic D., Schwartz G.J., Landmann R., Langhans W. (2004). Role for CD14, TLR2, and TLR4 in bacterial product-induced anorexia. Am. J. Physiol. Regul. Integr. Comp. Physiol..

[83] Altan Ö., Pabuçcuoğlu A., Altan A., Konyalioğlu S., Bayraktar H. (2003). Effect of heat stress on oxidative stress, lipid peroxidation and some stress parameters in broilers. Br. Poult. Sci..

[84] Kumar B. (2012). Stress and its impact on farm animals. Front. Biosci..

[85] Santos R.R., Awati A., Roubos-van den Hil P.J., Tersteeg-Zijderveld M.H.G., Koolmees P.A., Fink-Gremmels J. (2015). Quantitative histo-morphometric analysis of heat-stress-related damage in the small intestines of broiler chickens. Avian Pathol..

[86] Ulluwishewa D., Anderson R.C., McNabb W.C., Moughan P.J., Wells J.M., Roy N.C. (2011). Regulation of tight junction permeability by intestinal bacteria and dietary components. J. Nutr..

[87] Tomasello G., Mazzola M., Leone A., Sinagra E., Zummo G., Farina F., Damiani P., Cappello F., Gerges Geagea A., Jurjus A. (2016). Nutrition, oxidative stress and intestinal dysbiosis: Influence of diet on gut microbiota in inflammatory bowel diseases. Biomed. Pap..

[88] Shehata A.A., Yalçın S., Latorre J.D., Basiouni S., Attia Y.A., Abd El-Wahab A., Visscher C., El-Seedi H.R., Huber C., Hafez H.M. (2022). Probiotics, prebiotics, and phytogenic substances for optimizing gut health in poultry. Microorganisms.

[89] Oleskin A.V., Shenderov B.A. (2016). Neuromodulatory effects and targets of the SCFAs and gasotransmitters produced by the human symbiotic microbiota. Microb. Ecol. Health Dis..

[90] Tse J.K.Y. (2017). Gut microbiota, nitric oxide, and microglia as prerequisites for neurodegenerative disorders. ACS Chem. Neurosci..

[91] Saint-Georges-Chaumet Y., Edeas M. (2016). Microbiota–mitochondria inter-talk: Consequence for microbiota–host interaction. Pathog. Dis..

[92] Winter S.E., Thiennimitr P., Winter M.G., Butler B.P., Huseby D.L., Crawford R.W., Russell J.M., Bevins C.L., Adams L.G., Tsolis R.M. (2010). Gut inflammation provides a respiratory electron acceptor for salmonella. Nature.

[93] Tsolis R.M., Bäumler A.J. (2020). Gastrointestinal host-pathogen interaction in the age of microbiome research. Curr. Opin. Microbiol..

[94] Faralli A., Shekarforoush E., Ajalloueian F., Mendes A.C., Chronakis I.S. (2019). In vitro permeability enhancement of curcumin across CACO-2 cells monolayers using electrospun xanthan-chitosan nanofibers. Carbohydr. Polym..

[95] Wang J., Ghosh S.S., Ghosh S. (2017). Curcumin Improves intestinal barrier function: Modulation of Intracellular signaling, and organization of tight junctions. Am. J. Physiol. Cell Physiol..

[96] Baumgard L.H., Rhoads R.P. (2013). Effects of heat stress on postabsorptive metabolism and energetics. Annu. Rev. Anim. Biosci..

[97] Pearce S.C., Mani V., Weber T.E., Rhoads R.P., Patience J.F., Baumgard L.H., Gabler N.K. (2013). Heat stress and reduced plane of nutrition decreases intestinal integrity and function in pigs. J. Anim. Sci..

[98] Gilani S., Chrystal P.V., Barekatain R. (2021). Current experimental models, assessment and dietary modulations of intestinal permeability in broiler chickens. Anim. Nutr..

[99] Kvidera S.K., Dickson M.J., Abuajamieh M., Snider D.B., Fernandez M.V.S., Johnson J.S., Keating A.F., Gorden P.J., Green H.B., Schoenberg K.M. (2017). Intentionally Induced intestinal barrier dysfunction causes inflammation, affects metabolism, and reduces productivity in lactating Holstein cows. J. Dairy Sci..

[100] Omotayo O.P., Omotayo A.O., Mwanza M., Babalola O.O. (2019). Prevalence of mycotoxins and their consequences on human health. Toxicol. Res..

[101] Murugesan G.R., Ledoux D.R., Naehrer K., Berthiller F., Applegate T.J., Grenier B., Phillips T.D., Schatzmayr G. (2015). Prevalence and effects of mycotoxins on poultry health and performance, and recent development in mycotoxin counteracting strategies. Poult. Sci..

[102] Liu M., Zhao L., Gong G., Zhang L., Shi L., Dai J., Han Y., Wu Y., Khalil M.M., Sun L. (2022). Invited review: Remediation strategies for mycotoxin control in feed. J. Anim. Sci. Biotechnol..

[103] Zhao L., Zhang L., Xu Z., Liu X., Chen L., Dai J., Karrow N.A., Sun L. (2021). Occurrence of aflatoxin B1, deoxynivalenol and zearalenone in feeds in China during 2018–2020. J. Anim. Sci. Biotechnol..

[104] Wang R.J., Fui S.X., Miao C.H., Feng D.Y. (2005). Effects of different mycotoxin adsorbents on performance, meat characteristics and blood profiles of avian broilers fed mold contaminated corn. Asian Australas. J. Anim. Sci..

[105] Ghareeb K., Awad W.A., Böhm J., Zebeli Q. (2015). Impacts of the feed contaminant deoxynivalenol on the intestine of monogastric animals: Poultry and swine: Effect of deoxynivalenol on gut health. J. Appl. Toxicol..

[106] Niemiec J., Borzemska W., Goliński P., Karpińska E., Szeleszczuk P., Celeda T. (1994). The effect of ochratoxin A on egg quality, development of embryos and the level of toxinin eggs and tissues of hens and chicks. J. Anim. Feed Sci..

[107] Longobardi C., Andretta E., Romano V., Lauritano C., Avantaggiato G., Schiavone A., Jarriyawattanachaikul W., Florio S., Ciarcia R., Damiano S. (2020). Effects of some new antioxidants on apoptosis and ROS production in AFB1 treated chickens. Med. Sci. Forum.

[108] Liu Y., Wang W. (2016). Aflatoxin B1 Impairs mitochondrial functions, activates ROS generation, induces apoptosis and involves Nrf2 signal pathway in primary broiler hepatocytes: AFB1 on apoptosis and Nrf2 pathway in PBHs. Anim. Sci. J..

[109] Wang W.-J., Xu Z.-L., Yu C., Xu X.-H. (2017). Effects of aflatoxin B1 on mitochondrial respiration, ROS generation and apoptosis in broiler cardiomyocytes. Anim. Sci. J..

[110] Ma Q., Li Y., Fan Y., Zhao L., Wei H., Ji C., Zhang J. (2015). Molecular mechanisms of lipoic acid protection against aflatoxin b₁-induced liver oxidative damage and inflammatory responses in broilers. Toxins.

[111] Maurya B.K., Trigun S.K. (2016). Fisetin modulates antioxidant enzymes and inflammatory factors to inhibit aflatoxin-B1 induced hepatocellular carcinoma in rats. Oxidative Med. Cell. Longev..

[112] da Silva E.O., Bracarense A.P.F.L., Oswald I.P. (2018). Mycotoxins and oxidative stress: Where are we?. World Mycotoxin J..

[113] Mousa S.A., Abdel-Raheem S.M., Abdel-Raheem H.A., Sadeek A.L.S. (2017). Effect of dietary fat sources and antioxidant types on growth performance and carcass quality of japanese quails. Int. J. Poult. Sci..

[114] Attia Y.A., Al-Harthi M.A., Abo El-Maaty H.M. (2020). The effects of different oil sources on performance, digestive enzymes, carcass traits, biochemical, immunological, antioxidant, and morphometric responses of broiler chicks. Front. Vet. Sci..

[115] Alagawany M., Elnesr S.S., Farag M.R., Abd El-Hack M.E., Khafaga A.F., Taha A.E., Tiwari R., Yatoo M.I., Bhatt P., Khurana S.K. (2019). Omega-3 and omega-6 Fatty acids in poultry nutrition: Effect on production performance and health. Animals.

[116] Labuza L.R., Dugan J.R. (1971). Kinetics of lipid oxidation in foods critical reviews in food science and nutrition. Crit. Rev. Food Sci. Nutr..

[117] St. Angelo A.J. (1992). Lipid Oxidation in Food.

[118] Goicoechea E., Brandon M.H., Blokland M.D. (2011). Guillén Fate in digestion in vitro of several food components, including some toxic compounds coming from omega-3 and omega-6 lipids. Food Chem. Toxicol..

[119] Goicoechea E., Guillén M.D. (2014). Volatile compounds generated in corn oil stored at room temperature. presence of toxic compounds. Eur. J. Lipid Sci. Technol..

[120] Grigorakis K., Giogios I., Vasilaki A., Nengas I. (2010). Effect of the fish oil, oxidation status and of heat treatment temperature on the volatile compounds of the produced fish feeds animal feed science and technology. Anim. Feed Sci. Technol..

[121] Hammouda I.B., Zribi A., Mansour A.B., Bouaziz M. (2017). Effect of deep-frying on 3-MCPD esters and glycidyl esters contents and quality control of refined olive pomace blended with refined palm oil. Eur. Food Res. Technol..

[122] Takahashi K., Akiba Y. (1999). Effect of oxidized fat on perfor mance and some physiological responses in broiler chickens. Jpn. Poult. Sci..

[123] Anjum M.I., Mirza I.H., Khan A.G., Azim A. (2004). Effect of fresh versus oxidized soybean oil on growth performance, or gans weights and meat quality of broiler chicks. Pakistan Vet. J..

[124] Tavárez M.A., Boler D.D., Bess K.N., Zhao J., Yan F., Dilger A.C., McKeith F.K., Killefer J. (2011). Effect of antioxidant inclusion and oil quality on broiler performance, meat quality, and lipid oxidation. Poult. Sci..

[125] Boler D.D., Fernández-Dueñas D.M., Kutzler L.W., Zhao J., Harrell R.J., Campion D.R., McKeith F.K., Killefer J., Dilger A.C. (2012). Effects of oxidized corn oil and a synthetic antioxidant blend on performance, oxidative status of tissues, and fresh meat quality in finishing barrows. J. Anim. Sci..

[126] Kalyanaraman B. (2013). Teaching the basics of redox biology to medical and graduate students: Oxidants, antioxidants and disease mechanisms. Redox Biol..

[127] Kanazawa K., Ashida H. (1998). Dietary hydroperoxides of linoleic acid decompose to aldehydes in stomach before being absorbed into the body. Biochim. Biophys. Acta.

[128] Engberg R.M., Lauridsen C., Jensen S.K., Jakobsen K. (1996). Inclusion of oxidized vegetable oil in broiler diets. its influence on nutrient balance and on the antioxidative status of broilers. Poult. Sci..

[129] Cho J.H., Kim H.J., Kim I.H. (2014). Effects of phytogenic feed additive on growth performance, digestibility, blood metabolites, intestinal microbiota, meat color and relative organ weight after oral challenge with *Clostridium perfringens* in broilers. Livest. Sci..

[130] Valenzuela-Grijalva N.V., Pinelli-Saavedra A., Muhlia-Almazan A., Domínguez-Díaz D., González-Ríos H. (2017). Dietary Inclusion effects of phytochemicals as growth promoters in animal production. J. Anim. Sci. Technol..

[131] Tellez-Isaias G., Latorre J.D. (2022). Editorial: Alternatives to antimicrobial growth promoters and their impact in gut microbiota, health and disease: Volume II. Front. Vet. Sci..

[132] Papuc C., Goran G.V., Predescu C.N., Nicorescu V. (2017). Mechanisms of oxidative processes in meat and toxicity induced by postprandial degradation products: A review. Compr. Rev. Food Sci. Food Saf..

[133] Sandoval-Acuña C., Ferreira J., Speisky H. (2014). Polyphenols and mitochondria: An update on their increasingly emerging ros-scavenging independent actions. Arch. Biochem. Biophys..

[134] Di Meo F., Lemaur V., Cornil J., Lazzaroni R., Duroux J.-L., Olivier Y., Trouillas P. (2013). Free radical scavenging by natural polyphenols: Atom versus electron transfer. J. Phys. Chem. A.

[135] Halliwell B. (2006). Reactive species and antioxidants. Redox Biology is a fundamental theme of aerobic life. Plant Physiol..

[136] Karamać M. (2009). Chelation of Cu(II), Zn(II), and Fe(II) by Tannin constituents of selected edible nuts. Int. J. Mol. Sci..

[137] Singhal S.S., Awasthi S., Pandya U., Piper J.T., Saini M.K., Cheng J.Z., Awasthi Y.C. (1999). The effect of curcumin on glutathione-linked enzymes in K562 human leukemia cells. Toxicol. Lett..

[138] Romeo L., Intrieri M., D’Agata V., Mangano N.G., Oriani G., Ontario M.L., Scapagnini G. (2009). The major green tea polyphenol, (-)-epigallocatechin-3-gallate, induces heme oxygenase in rat neurons and acts as an effective neuroprotective agent against oxidative stress. J. Am. Coll. Nutr..

[139] Procházková D., Boušová I., Wilhelmová N. (2011). Antioxidant and prooxidant properties of flavonoids. Fitoterapia.

[140] Surai P.F. (2014). Polyphenol compounds in the chicken/animal diet: From the past to the future. J. Anim. Physiol. Anim. Nutr..

[141] Lipiński K., Mazur M., Antoszkiewicz Z., Purwin C. (2017). Polyphenols in monogastric nutrition—A review. Ann. Anim. Sci..

[142] Paszkiewicz M., Budzyńska A., Różalska B., Sadowska B. (2012). The immunomodulatory role of plant polyphenols. Postepy Hig. Med. Dosw..

[143] Surai P.F. (2020). Antioxidants in poultry nutrition and reproduction: An update. Antioxidants.

[144] Saeed M., Naveed M., Arain M.A., Arif M., Abd El-Hack M.E., Alagawany M., Siyal F.A., Soomro R.N., Sun C. (2017). Quercetin: Nutritional and beneficial effects in poultry. World’s Poult. Sci. J..

[145] Silvestro S., Bramanti P., Mazzon E. (2021). Role of quercetin in depressive-like behaviors: Findings from animal models. Appl. Sci..

[146] Gao X., Xiao Z.-H., Liu M., Zhang N.-Y., Khalil M.M., Gu C.-Q., Qi D.-S., Sun L.-H. (2018). Dietary silymarin supplementation alleviates zearalenone-induced hepatotoxicity and reproductive toxicity in rats. J. Nutr..

[147] Chowdhury S., Mandal G.P., Patra A.K., Kumar P., Samanta I., Pradhan S. (2018). Different essential oils in diets of broiler chickens: 2. gut microbes and morphology, immune response, and some blood profile and antioxidant enzymes. Anim. Feed Sci. Technol..

[148] Farahat M.H., Abdallah F.M., Ali H.A., Hernandez-Santana A. (2017). Effect of dietary supplementation of grape seed extract on the growth performance, lipid profile, antioxidant status and immune response of broiler chickens. Animal.

[149] Chen Y., Chen H., Li W., Miao J., Chen N., Shao X., Cao Y. (2018). Polyphenols in *Eucalyptus* leaves improved the egg and meat qualities and protected against ethanol-induced oxidative damage in laying hens. J. Anim. Physiol. Anim. Nutr..

[150] He S., Li S., Arowolo M.A., Yu Q., Chen F., Hu R., He J. (2019). Effect of resveratrol on growth performance, rectal temperature and serum parameters of yellow-feather broilers under heat stress. Anim. Sci. J..

[151] Liu L.L., He J.H., Xie H.B., Yang Y.S., Li J.C., Zou Y. (2014). Resveratrol induces antioxidant and heat shock protein mRNA expression in response to heat stress in black-boned chickens. Poult. Sci..

[152] Saracila M., Panaite T.D., Soica C., Tabuc C., Olteanu M., Predescu C., Rotar M.C., Criste R.D. (2019). Use of a hydroalcoholic extract of Salix alba L. bark powder in diets of broilers exposed to high heat stress. S. Afr. J. Anim. Sci..

[153] Wang D., Huang H., Zhou L., Li W., Zhou H., Hou G., Liu J., Hu L. (2015). Effects of dietary supplementation with turmeric rhizome extract on growth performance, carcass characteristics, antioxidant capability, and meat quality of wenchang broiler chickens. Ital. J. Anim. Sci..

[154] Yang J.Y., Zhang H.J., Wang J., Wu S.G., Yue H.Y., Jiang X.R., Qi G.H. (2017). Effects of dietary grape proanthocyanidins on the growth performance, jejunum morphology and plasma biochemical indices of broiler chicks. Animal.

[155] Kapczynski D.R., Afonso C.L., Miller P.J. (2013). Immune responses of poultry to Newcastle disease virus. Dev. Comp. Immunol..

[156] Bafundo K.W., Johnson A.B., Mathis G.F. (2020). The effects of a combination of quillaja saponaria and yucca schidigera on eimeria spp. in broiler chickens. Avian Dis..

[157] Li T., Yu H., Song Y., Zhang R., Ge M. (2019). Protective effects of ganoderma triterpenoids on cadmium-induced oxidative stress and inflammatory injury in chicken livers. J. Trace Elem. Med. Biol..

[158] Sandner G., Mueller A.S., Zhou X., Stadlbauer V., Schwarzinger B., Schwarzinger C., Wenzel U., Maenner K., van der Klis J.D., Hirtenlehner S. (2020). Ginseng extract ameliorates the negative physiological effects of heat stress by supporting heat shock response and improving intestinal barrier integrity: Evidence from studies with heat-stressed Caco-2 cells, c. elegans and growing broilers. Molecules.

[159] Lai M.M.C., Zhang H.A., Kitts D.D. (2021). Ginseng prong added to broiler diets reduces lipid peroxidation in refrigerated and frozen stored poultry meats. Molecules.

[160] Kim Y.-J., Lee G.-D., Choi I.-H. (2014). Effects of dietary supplementation of red ginseng marc and α-tocopherol on the growth performance and meat quality of broiler chicken: Effects of red ginseng marc and α-tocopherol. J. Sci. Food Agric..

[161] Song Z., Xie K., Zhang Y., Xie Q., He X., Zhang H. (2021). Effects of dietary ginsenoside rg1 supplementation on growth performance, gut health, and serum immunity in broiler chickens. Front. Nutr..

[162] Chung T., Choi I. (2016). Growth performance and fatty acid profiles of broilers given diets supplemented with fermented red ginseng marc powder combined with Red Koji. Rev. Bras. Cienc. Avic..

[163] Mao J., Wang Y., Wang W., Duan T., Yin N., Guo T., Guo H., Liu N., An X., Qi J. (2022). Effects of Taraxacum Mongolicum Hand.-Mazz. (Dandelion) on growth performance, expression of genes coding for tight junction protein and mucin, microbiota composition and short chain fatty acids in ileum of broiler chickens. BMC Vet. Res..

[164] Yener Y., Yalçin S., Çolpan İ. (2020). Effects of dietary supplementation of red ginseng root powder on performance, immune system, cecal microbial population and some blood parameters in broilers. Ank. Üniversitesi Vet. Fakültesi Derg..

[165] Almeida-da-Silva C.L.C., Sivakumar N., Asadi H., Chang-Chien A., Qoronfleh M.W., Ojcius D.M., Essa M.M. (2022). Effects of frankincense compounds on infection, inflammation, and oral health. Molecules.

[166] Mohamed S.H., Attia A.I., Reda F.M., Abd El-Hack M.E., Ismail I.E. (2021). Impacts of dietary supplementation of *boswellia serrata* on growth, nutrients digestibility, immunity, antioxidant status, carcase traits and caecum microbiota of broilers. Ital. J. Anim. Sci..

[167] Al-Yasiry A.R.M., Kiczorowska B., Samolińska W., Kowalczuk-Vasilev E., Kowalczyk-Pecka D. (2017). The effect of boswellia serrata resin diet supplementation on production, hematological, biochemical and immunological parameters in broiler chickens. Animal.

[168] Guerrini A., Dalmonte T., Lupini C., Andreani G., Salaroli R., Quaglia G., Zannoni A., Scozzoli M., Forni M., Isani G. (2022). Influence of dietary supplementation with boswellia serrata and salix alba on performance and blood biochemistry in free-range leghorn laying hens. Vet. Sci..

[169] Konieczka P., Szkopek D., Kinsner M., Fotschki B., Juśkiewicz J., Banach J. (2020). Cannabis-derived cannabidiol and nanoselenium improve gut barrier function and affect bacterial enzyme activity in chickens subjected to *C. perfringens* challenge. Vet. Res..

[170] Tanney C.A.S., Backer R., Geitmann A., Smith D.L. (2021). Cannabis glandular trichomes: A cellular metabolite factory. Front. Plant Sci..

[171] Nagarkatti P., Pandey R., Rieder S.A., Hegde V.L., Nagarkatti M. (2009). Cannabinoids as Novel Anti-Inflammatory Drugs. Future Med. Chem..

[172] Vispute M.M., Sharma D., Mandal A.B., Rokade J.J., Tyagi P.K., Yadav A.S. (2019). Effect of dietary supplementation of hemp (*Cannabis Sativa*) and dill seed (*Anethum Graveolens*) on performance, serum biochemicals and gut health of broiler chickens. J. Anim. Physiol. Anim. Nutr..

[173] Zamljen T., Jakopič J., Hudina M., Veberič R., Slatnar A. (2021). Influence of intra and inter species variation in chilies (*Capsicum* spp.) on metabolite composition of three fruit segments. Sci. Rep..

[174] Cho S.-Y., Kim H.-W., Lee M.-K., Kim H.-J., Kim J.-B., Choe J.-S., Lee Y.-M., Jang H.-H. (2020). Antioxidant and Anti-inflammatory activities in relation to the flavonoids composition of pepper (*Capsicum Annuum* L.). Antioxidants.

[175] Pérez-González A., Prejanò M., Russo N., Marino T., Galano A. (2020). Capsaicin, a powerful •OH-inactivating ligand. Antioxidants.

[176] Cheng J., Lin Y., Tang D., Yang H., Liu X. (2022). Structural and gelation properties of five polyphenols-modified pork myofibrillar protein exposed to hydroxyl radicals. LWT.

[177] Liu Y., Song M., Che T.M., Bravo D., Pettigrew J.E. (2012). Anti-inflammatory effects of several plant extracts on porcine alveolar macrophages in vitro. J. Anim. Sci..

[178] Mendivil E.J., Sandoval-Rodriguez A., Meza-Ríos A., Zuñiga-Ramos L., Dominguez-Rosales A., Vazquez-Del Mercado M., Sanchez-Orozco L., Santos-Garcia A., Armendariz-Borunda J. (2019). Capsaicin induces a protective effect on gastric mucosa along with decreased expression of inflammatory molecules in a gastritis model. J. Funct. Foods.

[179] Liu S.J., Wang J., He T.F., Liu H.S., Piao X.S. (2021). Effects of natural capsicum extract on growth performance, nutrient utilization, antioxidant status, immune function, and meat quality in broilers. Poult. Sci..

[180] Zheng J., Zheng S., Feng Q., Zhang Q., Xiao X. (2017). Dietary capsaicin and its anti-obesity potency: From mechanism to clinical implications. Biosci. Rep..

[181] Liu J.G., Xia W.G., Chen W., Abouelezz K.F.M., Ruan D., Wang S., Zhang Y.N., Huang X.B., Li K.C., Zheng C.T. (2021). Effects of capsaicin on laying performance, follicle development, and ovarian antioxidant capacity in aged laying ducks. Poult. Sci..

[182] Ali A., Ponnampalam E.N., Pushpakumara G., Cottrell J.J., Suleria H.A.R., Dunshea F.R. (2021). Cinnamon: A natural feed additive for poultry health and production—A review. Animals.

[183] Rao P.V., Gan S.H. (2014). Cinnamon: A multifaceted medicinal plant. Evid.-Based Complementary Altern. Med..

[184] Pannee C., Wacharee L., Chandhanee I. (2014). Antiinflammatory effects of essential oil from the leaves of *Cinnamomum cassia* and cinnamaldehyde on lipopolysaccharide-stimulated J774A.1 Cells. J. Adv. Pharm. Technol. Res..

[185] Lillehoj H.S., Kim D.K., Bravo D.M., Lee S.H. (2011). Effects of dietary plant-derived phytonutrients on the genome-wide profiles and coccidiosis resistance in the broiler chickens. BMC Proc..

[186] Lee S.H., Lillehoj H.S., Jang S.I., Lee K.W., Park M.S., Bravo D., Lillehoj E.P. (2011). Cinnamaldehyde enhances in vitro parameters of immunity and reduces in vivo infection against avian coccidiosis. Br. J. Nutr..

[187] Burt S. (2004). Essential Oils: Their Antibacterial Properties and Potential Applications in Foods—A Review. Int. J. Food Microbiol..

[188] Bober Z., Stępień A., Aebisher D., Ożog Ł., Bartusik-Aebisher D. (2018). medicinal benefits from the use of black pepper, curcuma and ginger. Eur. J. Clin. Exp. Med..

[189] Karami M., Alimon A.R., Sazili A.Q., Goh Y.M., Ivan M. (2011). Effects of dietary antioxidants on the quality, fatty acid profile, and lipid oxidation of longissimus muscle in kacang goat with aging time. Meat Sci..

[190] Hernandez-Patlan D., Solís-Cruz B., Patrin Pontin K., Latorre J.D., Baxter M.F.A., Hernandez-Velasco X., Merino-Guzman R., Méndez-Albores A., Hargis B.M., Lopez-Arellano R. (2019). Evaluation of the dietary supplementation of a formulation containing ascorbic acid and a solid dispersion of curcumin with boric acid against salmonella enteritidis and necrotic enteritis in broiler chickens. Animals.

[191] Leyva-Diaz A.A., Hernandez-Patlan D., Solis-Cruz B., Adhikari B., Kwon Y.M., Latorre J.D., Hernandez-Velasco X., Fuente-Martinez B., Hargis B.M., Lopez-Arellano R. (2021). Evaluation of curcumin and copper acetate against salmonella typhimurium infection, intestinal permeability, and cecal microbiota composition in broiler chickens. J. Anim. Sci. Biotechnol..

[192] Solis-Cruz B., Hernandez-Patlan D., Petrone V.M., Pontin K.P., Latorre J.D., Beyssac E., Hernandez-Velasco X., Merino-Guzman R., Arreguin M.A., Hargis B.M. (2019). Evaluation of a *Bacillus*-based direct-fed microbial on aflatoxin b1 toxic effects, performance, immunologic status, and serum biochemical parameters in broiler chickens. Avian Dis..

[193] Petrone-Garcia V.M., Lopez-Arellano R., Patiño G.R., Rodríguez M.A.C., Hernandez-Patlan D., Solis-Cruz B., Hernandez-Velasco X., Alba-Hurtado F., Vuong C.N., Castellanos-Huerta I. (2021). Curcumin reduces enteric isoprostane 8-Iso-PGF2α and prostaglandin GF2α in Specific pathogen-free leghorn chickens challenged with *Eimeria maxima*. Sci. Rep..

[194] Lee S.H., Lillehoj H.S., Hong Y.H., Jang S.I., Lillehoj E.P., Ionescu C., Mazuranok L., Bravo D. (2010). In vitro effects of plant and mushroom extracts on immunological function of chicken lymphocytes and macrophages. Br. Poult. Sci..

[195] Liu Y., Song M., Che T.M., Bravo D., Maddox C.W., Pettigrew J.E. (2014). Effects of *Capsicum oleoresin*, garlic botanical, and turmeric oleoresin on gene expression profile of ileal mucosa in weaned pigs. J. Anim. Sci..

[196] Liu Y., Song M., Che T.M., Lee J.J., Bravo D., Maddox C.W., Pettigrew J.E. (2014). Dietary plant extracts modulate gene expression profiles in ileal mucosa of weaned pigs after an *Escherichia coli* infection. J. Anim. Sci..

[197] Liu Y., Che T.M., Song M., Lee J.J., Almeida J.a.S., Bravo D., Van Alstine W.G., Pettigrew J.E. (2013). Dietary plant extracts improve immune responses and growth efficiency of pigs experimentally infected with porcine reproductive and respiratory syndrome virus. J. Anim. Sci..

[198] Yadav S., Teng P.-Y., Souza dos Santos T., Gould R.L., Craig S.W., Lorraine Fuller A., Pazdro R., Kim W.K. (2020). The effects of different doses of curcumin compound on growth performance, antioxidant status, and gut health of broiler chickens challenged with *Eimeria species*. Poult. Sci..

[199] Xie Z., Shen G., Wang Y., Wu C. (2019). Curcumin supplementation regulates lipid metabolism in broiler chickens. Poult. Sci..

[200] Grzanna R., Lindmark L., Frondoza C.G. (2005). Ginger—An herbal medicinal product with broad anti-inflammatory actions. J. Med. Food.

[201] Stoner G.D. (2013). Ginger: Is it ready for prime time?. Cancer Prev. Res..

[202] Nile S.H., Park S.W. (2015). Chromatographic analysis, antioxidant, anti-inflammatory, and xanthine oxidase inhibitory activities of ginger extracts and its reference compounds. Ind. Crops Prod..

[203] Zhang M., Viennois E., Prasad M., Zhang Y., Wang L., Zhang Z., Han M.K., Xiao B., Xu C., Srinivasan S. (2016). Edible Ginger-Derived Nanoparticles: A novel therapeutic approach for the prevention and treatment of inflammatory bowel disease and colitis-associated cancer. Biomaterials.

[204] Kumar N.V., Murthy P.S., Manjunatha J.R., Bettadaiah B.K. (2014). Synthesis and quorum sensing inhibitory activity of key phenolic compounds of ginger and their derivatives. Food Chem..

[205] Citronberg J., Bostick R., Ahearn T., Turgeon D.K., Ruffin M.T., Djuric Z., Sen A., Brenner D.E., Zick S.M. (2013). Effects of ginger supplementation on cell-cycle biomarkers in the normal-appearing colonic mucosa of patients at increased risk for colorectal cancer: Results from a pilot, randomized, and controlled trial. Cancer Prev. Res..

[206] Karangiya V.K., Savsani H.H., Patil S.S., Garg D.D., Murthy K.S., Ribadiya N.K., Vekariya S.J. (2016). Effect of dietary supplementation of garlic, ginger and their combination on feed intake, growth performance and economics in commercial broilers. Vet. World.

[207] Abd El-Hack M.E., Alagawany M., Shaheen H., Samak D., Othman S.I., Allam A.A., Taha A.E., Khafaga A.F., Arif M., Osman A. (2020). Ginger and its derivatives as promising alternatives to antibiotics in poultry feed. Animals.

[208] Zhao X., Yang Z.B., Yang W.R., Wang Y., Jiang S.Z., Zhang G.G. (2011). Effects of ginger root (*Zingiber officinale*) on laying performance and antioxidant status of laying hens and on dietary oxidation stability. Poult. Sci..

[209] Kikuzaki H., Nakatani N. (1996). Cyclic diarylheptanoids from rhizomes of *Zingiber officinale*. Phytochemistry.

[210] Fuhrman B., Rosenblat M., Hayek T., Coleman R., Aviram M. (2000). Ginger extract consumption reduces plasma cholesterol, inhibits ldl oxidation and attenuates development of atherosclerosis in atherosclerotic, apolipoprotein E-deficient mice. J. Nutr..

[211] An S., Liu G., Guo X., An Y., Wang R. (2019). Ginger Extract Enhances Antioxidant Ability and Immunity of Layers. Anim. Nutr..

[212] Karthikeyan J., Rani P. (2003). Enzymatic and non-enzymatic antioxidants in selected piper species. Indian J. Exp. Biol..

[213] Abou-Elkhair R., Ahmed H.A., Selim S. (2014). Effects of black pepper (*Piper Nigrum*), turmeric powder (*Curcuma Longa*) and coriander seeds (*Coriandrum sativum*) and their combinations as feed additives on growth performance, carcass traits, some blood parameters and humoral immune response of broiler chickens. Asian-Australas. J. Anim Sci..

[214] Khalaf N.A., Shakya A., Al-Othman A., Elagbar Z., Farah H.S. (2008). Antioxidant activity of some common plants. Turk. J. Biol..

[215] Reen R.K., Roesch S.F., Kiefer F., Wiebel F.J., Singh J. (1996). Piperine impairs cytochrome P4501A1 activity by direct interaction with the enzyme and not by down regulation of *CYP1A1* gene expression in the rat hepatoma 5L cell line. Biochem. Biophys. Res. Commun..

[216] Malini T., Arunakaran J., Aruldhas M.M., Govindarajulu P. (1999). Effects of piperine on the lipid composition and enzymes of the pyruvate-malate cycle in the testis of the rat in vivo. Biochem. Mol. Biol. Int..

[217] Moorthy M., Ravi S., Ravikumar M., Viswanathan K., Edwin S.C. (2009). Ginger, pepper and curry leaf powder as feed additives in broiler diet. Int. J. Poult. Sci..

[218] Reynoso-Moreno I., Najar-Guerrero I., Escareño N., Flores-Soto M.E., Gertsch J., Viveros-Paredes J.M. (2017). An endocannabinoid uptake inhibitor from black pepper exerts pronounced anti-inflammatory effects in mice. J. Agric. Food Chem..

[219] Lee W., Yoo H., Kim J.A., Lee S., Jee J.-G., Lee M.M.Y., Lee Y.-M., Bae J.-S. (2013). Barrier protective effects of piperlonguminine in LPS-induced inflammation in vitro and in vivo. Food Chem. Toxicol..

[220] Ginzburg S., Golovine K.V., Makhov P.B., Uzzo R.G., Kutikov A., Kolenko V.M. (2014). Piperlongumine inhibits NF-ΚB activity and attenuates aggressive growth characteristics of prostate cancer cells. Prostate.

[221] Ghazalah A.A., El-Hakim A.S.A., Refaie A.M. (2007). Response of broiler chicks to some dietary growth promoters throughout different growth period. Egypt. Poult. Sci. J..

[222] Tollba A.A.H., Azouz H.M.M., El-Samad A.M.H. (2007). Antioxidants supplementation to diet of egyptian chicken under different environmental condition: 2-The Growth during cold winter stress. Egypt. Poult. Sci. J..

[223] Mansoub N.H. (2011). Comparison of using different level of black pepper with probiotic on performance and serum composition of broiler chickens. J. Basic Appl. Sci. Res..

[224] Akbarian A. (2012). influence of turmeric rhizome and black pepper on blood constituents and performance of broiler chickens. Afr. J. Biotechnol..

[225] Al-Kassie G.A.M., Butris G.Y., Ajeena S.J. (2012). The potency of feed supplemented mixture of hot red pepper and black pepper on the performance and some hematological blood traits on broiler diet. Int. J. Adv. Biol. Res..

[226] Tawfeek N., Mahmoud M.F., Hamdan D.I., Sobeh M., Farrag N., Wink M., El-Shazly A.M. (2021). Phytochemistry, pharmacology and medicinal uses of plants of the genus *Salix*: An updated review. Front. Pharmacol..

[227] Al-fataftah A., Abdelqader A. (2013). Effect of *Salix babylonica*, *Populus nigra* and *Eucalyptus camaldulensis* extracts in drinking water on performance and heat tolerance of broiler chickens during heat stress. Am.-Eurasian J. Agric. Environ. Sci..

[228] Panaite T.D., Saracila M., Papuc C.P., Predescu C.N., Soica C. (2020). Influence of dietary supplementation of salix alba bark on performance, oxidative stress parameters in liver and gut microflora of broilers. Animals.

[229] Saracila M., Tabuc C., Panaite T.D., Papuc C.P., Olteanu M., Criste R.D. (2018). Effect of the dietary willow bark extract (*Salix alba*) on the caecal microbial population of broilers (14–28 Days) reared at 32 °C. Agric. Life Life Agric. Conf. Proc..

[230] Yalçin S., Eser H., Onbaşilar İ., Yalçin S. (2020). Effects of dried thyme (*Thymus vulgaris* L.) leaves on performance, some egg quality traits and immunity in laying hens. Ank. Üniversitesi Vet. Fakültesi Derg..

[231] Ocaña A., Reglero G. (2012). Effects of thyme extract oils (from *Thymus Vulgaris*, *Thymus Zygis* and *Thymus hyemalis*) on cytokine production and gene expression of OxLDL-stimulated THP-1-macrophages. J. Obes..

[232] Amirghofran Z., Ahmadi H., Karimi M.H., Kalantar F., Gholijani N., Malek-Hosseini Z. (2016). In vitro inhibitory effects of thymol and carvacrol on dendritic cell activation and function. Pharm. Biol..

[233] Liang D., Li F., Fu Y., Cao Y., Song X., Wang T., Wang W., Guo M., Zhou E., Li D. (2014). Thymol inhibits LPS-stimulated inflammatory response via down-regulation of NF-ΚB and MAPK signaling pathways in mouse mammary epithelial cells. Inflammation.

[234] Hassan F.A.M., Awad A. (2017). Impact of thyme powder (*Thymus vulgaris* L.) supplementation on gene expression profiles of cytokines and economic efficiency of broiler diets. Environ. Sci. Pollut. Res. Int..

[235] Lee K.W., Everts H., Kappert H.J., Frehner M., Losa R., Beynen A.C. (2003). Effects of Dietary essential oil components on growth performance, digestive enzymes and lipid metabolism in female broiler chickens. Br. Poult. Sci..

[236] Shehata A.A., Attia Y., Khafaga A.F., Farooq M.Z., El-Seedi H.R., Eisenreich W., Tellez-Isaias G. (2022). Restoring healthy gut microbiome in poultry using alternative feed additives with particular attention to phytogenic substances: Challenges and prospects. Ger. J. Vet. Res..

[237] Říha M., Karlíčková J., Filipský T., Macáková K., Rocha L., Bovicelli P., Silvestri I.P., Saso L., Jahodář L., Hrdina R. (2014). In vitro evaluation of copper-chelating properties of flavonoids. RSC Adv..

[238] Papuc C., Goran G.V., Predescu C.N., Nicorescu V., Stefan G. (2017). Plant polyphenols as antioxidant and antibacterial agents for shelf-life extension of meat and meat products: Classification, structures, sources, and action mechanisms: Polyphenols extending meat shelf-life. Compr. Rev. Food Sci. Food Saf..

[239] Teng H., Chen L. (2019). Polyphenols and bioavailability: An update. Crit. Rev. Food Sci. Nutr..

[240] Scalbert A., Manach C., Morand C., Rémésy C., Jiménez L. (2005). Dietary polyphenols and the prevention of diseases. Crit. Rev. Food Sci. Nutr..

[241] Natella F., Nardini M., Giannetti I., Dattilo C., Scaccini C. (2002). Coffee drinking influences plasma antioxidant capacity in humans. J. Agric. Food Chem..

[242] Michiels J., Missotten J., Dierick N., Fremaut D., Maene P., De Smet S. (2008). In vitro degradation and in vivo passage kinetics of carvacrol, thymol, eugenol and *trans*-cinnamaldehyde along the gastrointestinal tract of piglets: In vitro degradation and in Vivo passage kinetics of essential oils in piglets. J. Sci. Food Agric..

[243] Stevanović Z.D., Bošnjak-Neumüller J., Pajić-Lijaković I., Raj J., Vasiljević M. (2018). Essential oils as feed additives—Future perspectives. Molecules.

[244] de Oliveira E.F., Paula H.C.B., de Paula R.C.M. (2014). Alginate/cashew gum nanoparticles for essential oil encapsulation. Colloids Surf. B Biointerfaces.

[245] Zhang Y., Gong J., Yu H., Guo Q., Defelice C., Hernandez M., Yin Y., Wang Q. (2014). Alginate-whey protein dry powder optimized for target delivery of essential oils to the intestine of chickens. Poult. Sci..

[246] Akbarzadeh A., Rezaei-Sadabady R., Davaran S., Joo S.W., Zarghami N., Hanifehpour Y., Samiei M., Kouhi M., Nejati-Koshki K. (2013). Liposome: Classification, preparation, and applications. Nanoscale Res. Lett..

[247] Iqbal M.F., Zhu W.-Y. (2009). Bioactivation of flavonoid diglycosides by chicken cecal bacteria. FEMS Microbiol. Lett..

